# New species of *Austropurcellia*, cryptic short-range endemic mite harvestmen (Arachnida, Opiliones, Cyphophthalmi) from Australia’s Wet Tropics biodiversity hotspot

**DOI:** 10.3897/zookeys.586.6774

**Published:** 2016-05-04

**Authors:** Katya R. Jay, Zachary R. Popkin-Hall, Michelle J. Coblens, Jill T. Oberski, Prashant P. Sharma, Sarah L. Boyer

**Affiliations:** 1 Macalester College, Saint Paul, United States of America; 2 Texas A&M University, College Station, United States of America; 3 University of Wisconsin, Madison, USA

**Keywords:** Queensland, rainforest, biogeography, morphology, taxonomy

## Abstract

The genus *Austropurcellia* is a lineage of tiny leaf-litter arachnids that inhabit tropical rainforests throughout the eastern coast of Queensland, Australia. The majority of their diversity is found within the Wet Tropics rainforests of northeast Queensland, an area known for its exceptionally high levels of biodiversity and endemism. Studying the biogeographic history of limited-dispersal invertebrates in the Wet Tropics can provide insight into the role of climatic changes such as rainforest contraction in shaping rainforest biodiversity patterns. Here we describe six new species of mite harvestmen from the Wet Tropics rainforests, identified using morphological data, and discuss the biogeography of *Austropurcellia* with distributions of all known species. With this taxonomic contribution, the majority of the known diversity of the genus has been documented.

## Introduction

**Figure 1.**
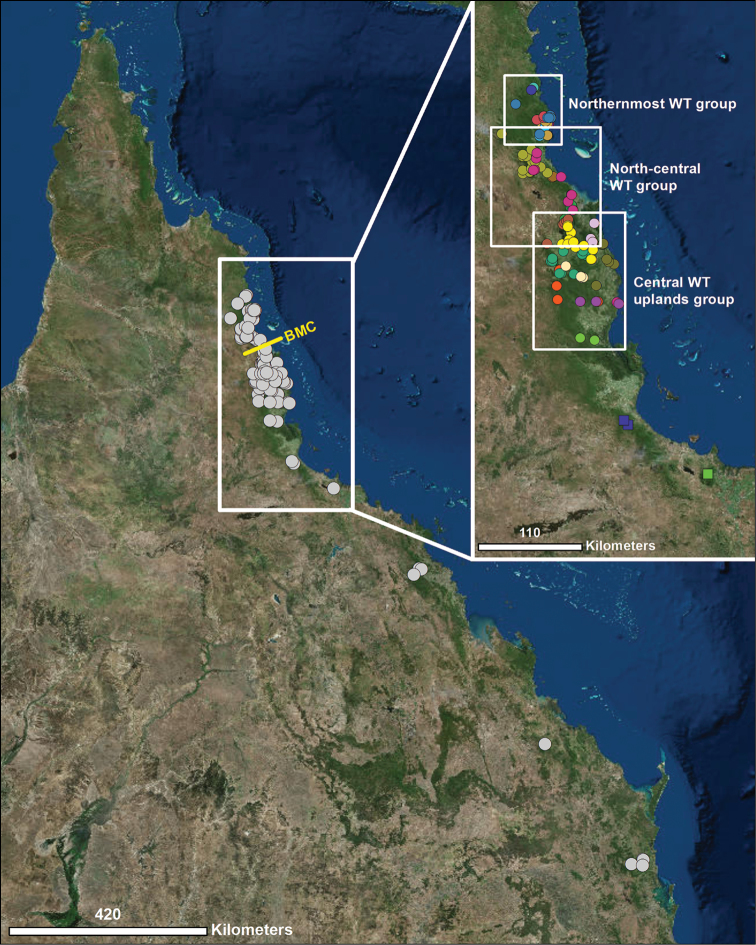
Distribution of all *Austropurcellia* species found throughout Queensland, Australia. Largest white box outlines the Wet Tropics World Heritage Area, shown in closer detail to the right. Smaller boxes within inset map represent groups of closely related species found within the Wet Tropics by Boyer et al. (2015), shown in larger detail in Figs 2, 3, & 4. Each circle denotes a locality and each colored circle denotes a different species found in one of the three relevant groups. Colored squares indicate the two species found in the southern Wet Tropics, which was excluded for the purposes of this study because it does not contain any of the new species presented.

**Figure 2.**
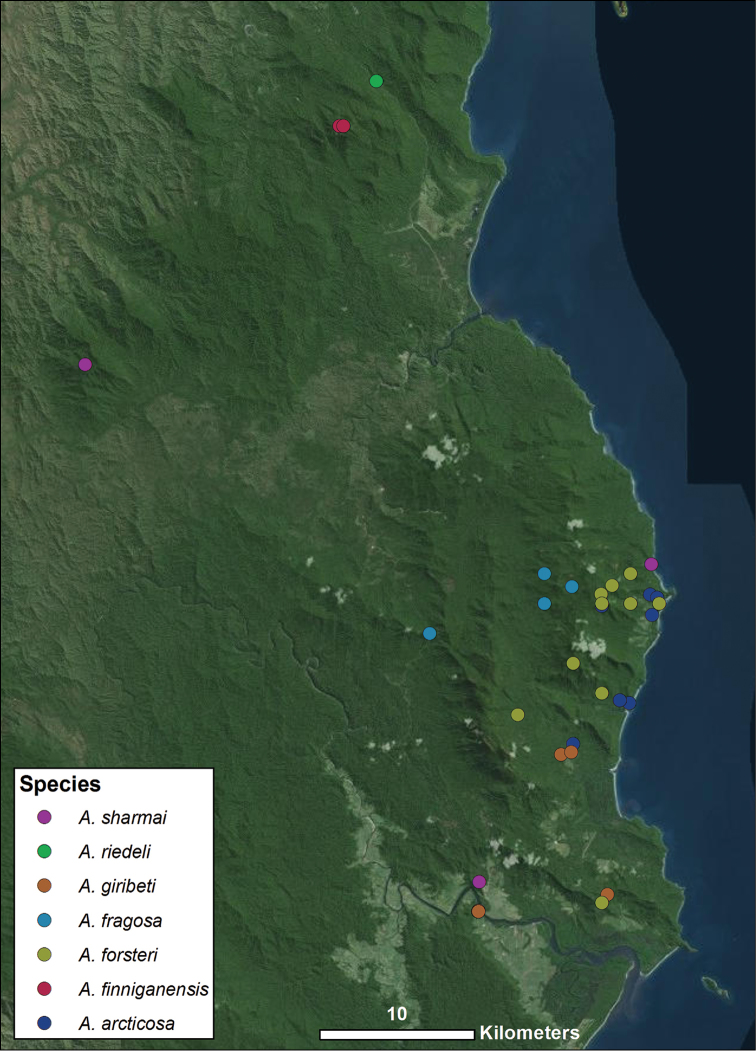
Distribution of all species found within northernmost WT group, corresponding with Fig. 1. Each colored icon denotes a different species, as indicated in the legend.

**Figure 3.**
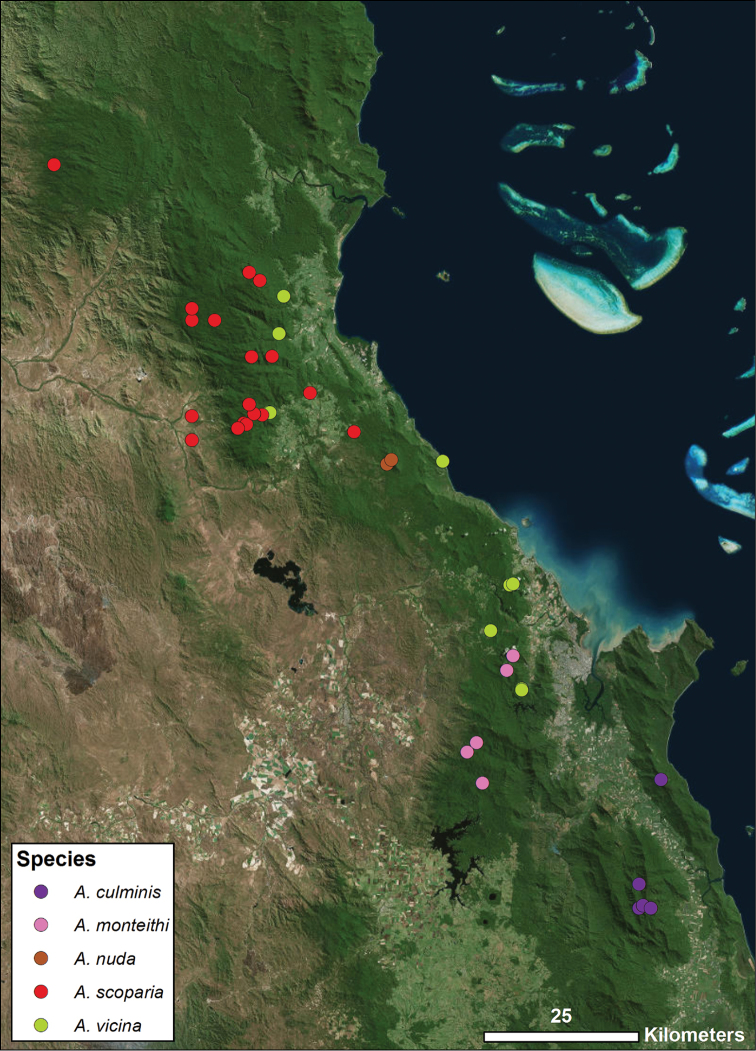
Distribution of all species found within north-central WT group, corresponding with Fig. 1. Each colored icon denotes a different species, as indicated in the legend.

**Figure 4.**
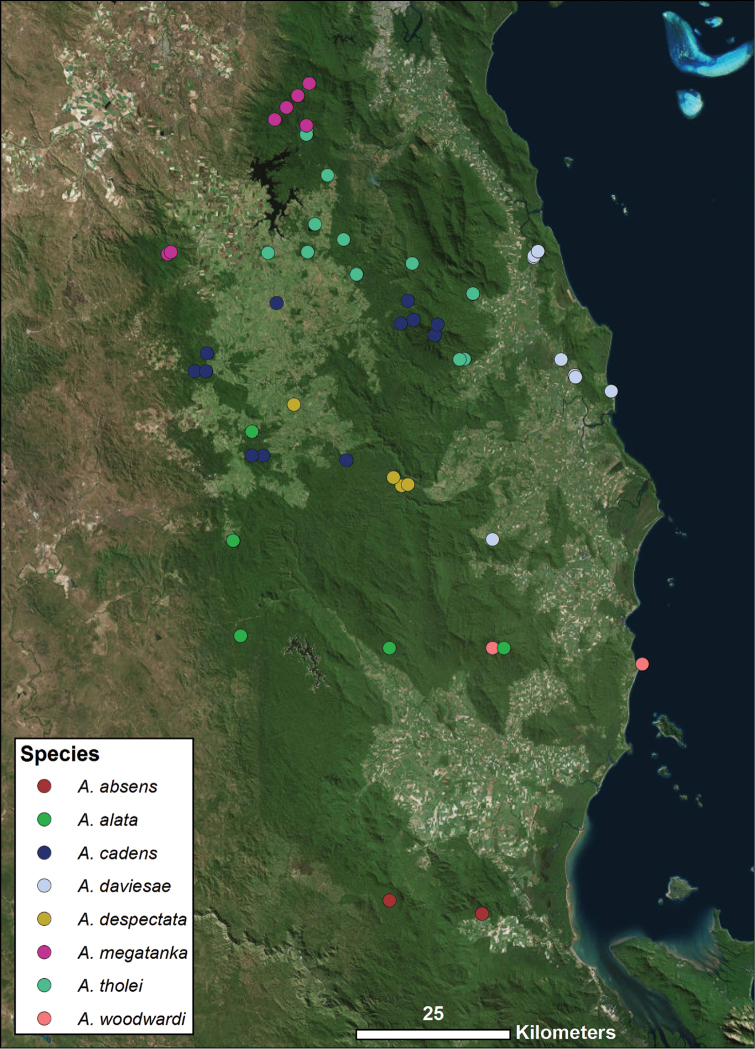
Distribution of all species found within central WT uplands group, corresponding with Fig. 1. Each colored icon denotes a different species, as indicated in the legend.

The mite harvestmen (order Opiliones, suborder Cyphophthalmi) are a globally distributed suborder of tiny (1.5–5 mm), cryptic arachnids that are extremely dispersal-limited, making them ideal for fine-scale historical biogeographic studies. Nearly all species are known from pristine leaf-litter habitats in tropical, subtropical, and temperate forests, with a few others from caves (Juberthie 1971, Giribet et al. 2012) . The mite harvestmen that are endemic to the Wet Tropics World Heritage Area (WT) of Queensland, in northeast Australia, are members of the genus *Austropurcellia* Juberthie, 1988, with a range spanning the WT in the north to the Queensland-New South Wales border in the south. The highest diversity of species is found in the rainforests of the WT (Figs 1–4). *Austropurcellia* is a member of the family Pettalidae Shear, 1980, a lineage with a classical temperate Gondwanan distribution that includes species from Chile, South Africa, Madagascar, Sri Lanka, Western Australia, and New Zealand (Boyer and Giribet 2007). Phylogenetic analyses of this group have demonstrated monophyly of all Queensland mite harvestmen (Boyer and Giribet 2007, Giribet et al. 2012, Boyer et al. 2015). Therefore, all Queensland species were transferred to *Austropurcellia* by Boyer and Giribet (2007), including species originally described as members of the genera *Neopurcellia* Forster, 1948 and *Rakaia* Hirst, 1925, whose type species occur in New Zealand.

Prior to 2012, only five *Austropurcellia* species were known (four from the WT and one from Central Queensland). Thus, little was known about the evolutionary history of the genus and its diversity in the region. Subsequently, Boyer and Reuter (2012) described four new species of mite harvestmen from the WT, and Popkin-Hall and Boyer (2014) described three new species from southeast Queensland. Boyer et al. (2015) presented six new species in a phylogenetic study of *Austropurcellia*, providing further insight into the historical biogeography of the genus. Together, these new species expanded *Austropurcellia*’s known range to cover most of Queensland’s coast. After intensive examination of museum collections and a series of collecting campaigns by the authors and collaborators, there are currently 19 described species within *Austropurcellia*, including 15 species from the WT alone.

*Austropurcellia* is an ancient lineage, and its evolutionary history has no doubt been shaped by the turbulent geological and climatic history of the Australian continent. Molecular dating suggests that *Austropurcellia* underwent initial diversification in the late Cretaceous (Giribet et al. 2012, Giribet et al. in press). Since then, the genus has persisted despite significant climatic changes in the region. Following the separation of the Australian continent from East Antarctica and the establishment of the Antarctic Circumpolar Current (ACC) in the Oligocene, global cooling occurred and latitudinal temperature gradients steepened (Crisp et al. 2004, Byrne et al. 2008). Australia drifted north into warmer latitudes, partially offsetting the cooling effects of the ACC, leading to a drier and more seasonal climate by the onset of the Miocene (~23 Ma). Rainforest habitats suitable for *Austropurcellia* were widespread throughout the Australian continent during the early Miocene, before they were largely replaced by sclerophyllous vegetation during a late Tertiary phase of long-term climate change and aridification (Adam 1992, Truswell 1993, Schneider et al. 1998, Crisp et al. 2004, Byrne et al. 2008). Miocene climatic changes have been invoked as a putative driver of speciation processes in other ancient Australian lineages. For example, phylogenetic and biogeographic analyses of Australian Archaeidae (assassin spiders), another limited-dispersal temperate Gondwanan arachnid group found in Queensland, point to evolutionary divergence as a result of Miocene aridification events (Rix and Harvey 2012). *Austropurcellia* provides a relevant point of comparison with this group, and work in preparation by the authors will examine tempo and age of speciation events within the genus.

The WT is considered to be a model system for studying biogeographic processes that shape rainforest diversity because it contains disproportionately large percentages of Australia’s fauna (despite comprising only 0.12% of the continent by area), as well as unusually high rates of endemism (Nix 1991, Williams 2006, Rix and Harvey 2012). Palynological records from the WT suggest that significant range contractions and expansions of forest habitats have occurred as a result of more recent climate change. In particular, angiosperm rainforests were replaced by sclerophyllous or drier gymnosperm-dominated forests during Pleistocene glacial and interglacial cycles prior to the establishment of the current climate (Kershaw 1994, Graham et al. 2006, Bell et al. 2007). Rainforests have persisted in some areas of the WT much more consistently than others, leading to identification of potential species refugia by Webb and Tracey (1981), which take the form of small upland rainforest fragments scattered throughout areas of warmer and drier habitats (Schneider et al. 1998, Graham et al. 2006, Graham et al. 2010). Mite harvestmen only need small patches of suitable habitat to persist, and are thus able to survive these severe rainforest contraction events, making them an ideal group to study historical biogeography and speciation in the WT (e.g. Boyer et al. 2005, 2007b, Clouse and Giribet 2007, 2010, Boyer and Giribet 2009, Giribet et al. 2012, Boyer et al. 2015). Boyer et al. (2016) modeled suitable climatic conditions for *Austropurcellia* and projected them onto paleoclimate data layers from time slices going back to the Last Glacial Maximum (LGM). They found that differences in LGM climatic suitability across the WT were a strong predictor of present-day diversity, outperforming current climatic suitability. This suggests that the LGM climatic refugia acted as museums of biodiversity, preserving lineages during a restrictive climatic regime and shaping the distribution of biodiversity across the WT that is seen today.

Mite harvestmen are known to have very low dispersal rates, with species even in well-surveyed areas generally found in only a few localities within a 50-km radius (Boyer and Giribet 2009, Boyer et al. 2015, Clouse et al. 2016) (Figs 1–4). Previous phylogenetic and biogeographic work has indicated that different closely related groups of species within *Austropurcellia* occupy distinct geographic areas of the WT. As examples, species north of the Black Mountain Corridor (BMC), an area that experienced loss of rainforest habitat during the Last Glacial Maximum, such as *Austropurcellia articosa* and *Austropurcellia giribeti*, form a distinct clade. *Austropurcellia* from the north-central WT and central WT uplands regions comprise another species group (Boyer et al. 2015) (Figs 1–4). Unique morphological features also tend to be shared between closely related species. Therefore, combining morphological and geographic data can provide reciprocally corroborative insights into the evolutionary history of the genus.

Here we present six new species of mite harvestmen from the WT that are morphologically distinct from other members of *Austropurcellia*. We identify several diagnostic characters that vary between groups of species whose ranges are geographically proximate, and use this information to form hypotheses about the new species’ phylogenetic relationships.

## Methods

Specimens were hand-collected by the authors and colleagues in the WT of Queensland, Australia by sifting leaf litter during 2011-2015 and preserved in 95% ethanol. Additional specimens were provided by collections from Harvard’s Museum of Comparative Zoology (MCZ), the Queensland Museum (QM), and the Australian National Insect Collection (ANIC). GPS data were recorded at each locality.

Collected specimens were examined for morphological differences using light microscopy and sorted into putative morphospecies. Due to their small size and highly conserved morphology, species-level differences are often only visible using an SEM. Therefore, males from different localities were examined on a scanning electron microscope (SEM). Only males possess characters that are diagnostic at the level of species.

Holotype specimens were photographed using an Olympus SZX10 light microscope driven by Leica Acquire software (Leica Microsystems) at multiple focal planes. Image series were integrated using Helicon Focus (Helicon Soft Limited). Specimens were placed in hand sanitizer for lateral images.

**Figure 5.**
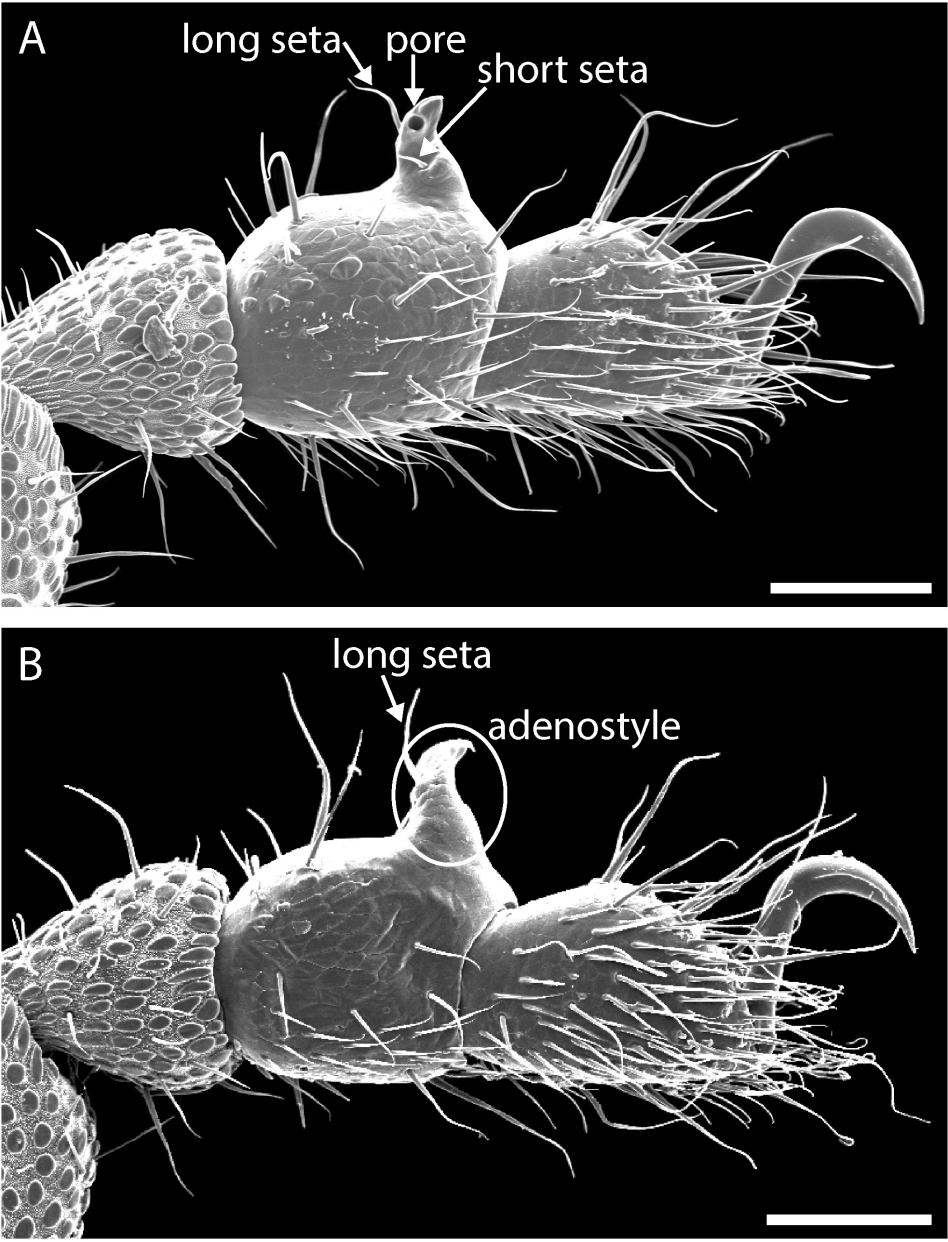
*Austropurcellia megatanka* sp. n., tarsus and metatarsus IV with diagnostic features labeled. **A** male tarsus and metatarsus IV, lateral view, showing adenostyle pore and setae **B** male tarsus and metatarsus IV, medial view, showing adenostyle and long seta. Scale bars: 100 μm.

Paratype males chosen for SEM were dissected under the light microscope and mounted on stubs. One of each walking leg (I-III) as well as one palp and one chelicera were removed and mounted on a single stub. Both legs IV were mounted to provide a lateral and medial view of distinguishing features. One female leg IV was also mounted for comparison. Males were mounted ventrally on another stub to allow for close examination of the anal plate and scopula, and remounted for examination of dorsal ornamentation. Stubs were coated with gold-palladium alloy using a Denton Vacuum Desk III sputter coater and imaged using a JEOL JSM-6610LV SEM. Appendage measurements were made using the digital scalar tool included in the JEOL software package. New species were diagnosed based on several key character systems that are demonstrably informative in *Austropurcellia* taxonomy: male anal plate shape, scopula size and shape, tarsus IV segmentation and shape, and adenostyle shape (Fig. 5) (Boyer and Reuter 2012, Boyer et al. 2015).

SEM images for new species were edited to have a uniform black background using Adobe Photoshop CS6 Extended and compiled into plates using Adobe Illustrator CS6. ArcGIS 10.2.1 was used to create distribution maps for species.

## Taxonomy

All material examined is from Queensland, Australia.

### Order OPILIONES Sundevall, 1833 Suborder CYPHOPHTHALMI Simon, 1879 Infraorder SCOPULOPHTHALMI Giribet, Sharma, Benavides, Boyer, Clouse, de Bivort, Dimitrov, Kawauchi, Murienne & Schwendinger, 2011 Family PETTALIDAE Shear, 1980

#### Genus Austropurcellia

Taxon classificationAnimaliaOpilionesPettalidae

Juberthie, 1988

##### Type species.

*Austropurcellia scoparia* Juberthie, 1988

##### Species included.

*Austropurcellia absens* Boyer & Popkin-Hall, 2015, *Austropurcellia acuta* Popkin-Hall & Boyer, 2014, *Austropurcellia alata* Boyer & Reuter, 2012, *Austropurcellia arcticosa* Cantrell, 1980, *Austropurcellia barbata* Popkin-Hall & Boyer, 2014, *Austropurcellia cadens* Baker & Boyer, 2015, *Austropurcellia capricornia* Todd Davies, 1977, *Austropurcellia clousei* Boyer, Baker & Popkin-Hall, 2015, *Austropurcellia culminis* Boyer & Reuter, 2012, *Austropurcellia daviesae* Juberthie, 1989, *Austropurcellia despectata* Boyer & Reuter, 2012, *Austropurcellia forsteri* Juberthie, 2000, *Austropurcellia giribeti* Boyer & Quay, 2015, *Austropurcellia scoparia* Juberthie, 1988, *Austropurcellia sharmai* Boyer & Quay, 2015, *Austropurcellia superbensis* Popkin-Hall & Boyer, 2014, *Austropurcellia tholei* Baker & Boyer, 2015, *Austropurcellia vicina* Boyer & Reuter, 2012, *Austropurcellia woodwardi* Forster, 1955.

#### Austropurcellia finniganensis

Taxon classificationAnimaliaOpilionesPettalidae

Popkin-Hall, Jay & Boyer sp. n.

http://zoobank.org/33A77AC4-9D33-4DAA-A0C7-E6FC0C6FE311

**Figure 6.**
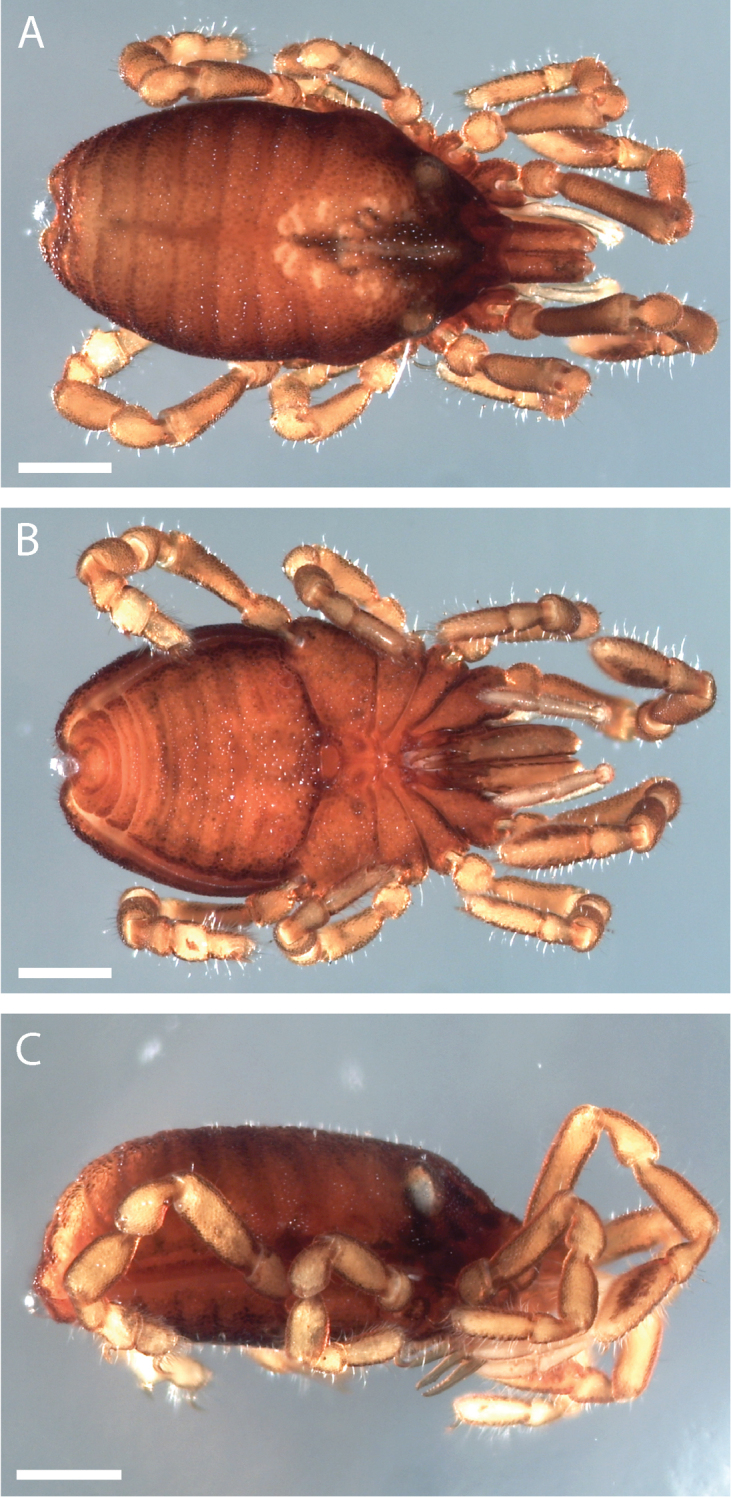
*Austropurcellia finniganensis* sp. n., holotype male, QM 102446. **A** dorsal view **B** ventral view **C** lateral view. Scale bars: 0.5 mm.

**Figure 7.**
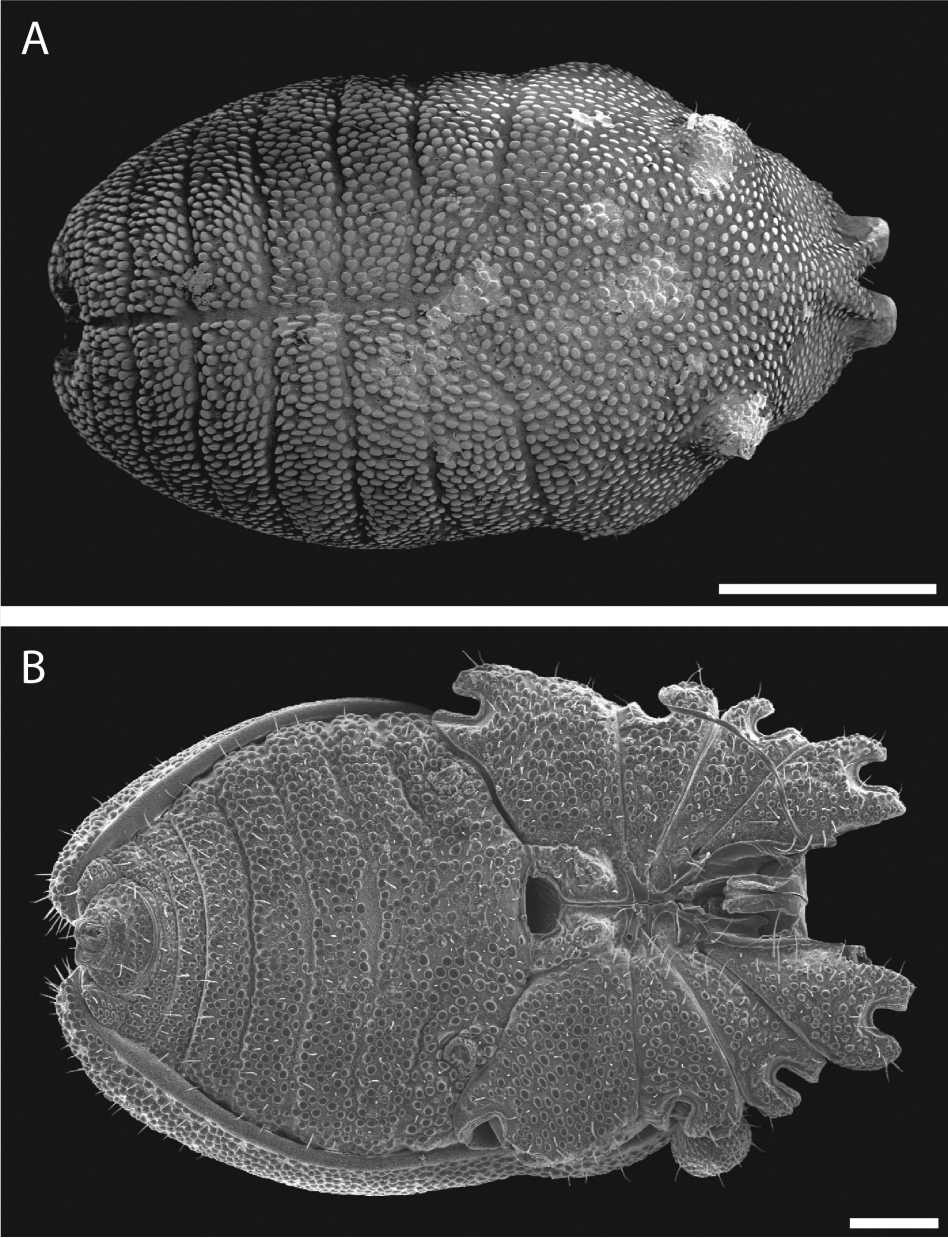
*Austropurcellia finniganensis* sp. n., males. **A** dorsal view, QM berlesate 981 **B** ventral view, QM 102447, paratype. Scale bar: 0.5 μm (**A**); 200 μm (**B**).

**Figure 8.**
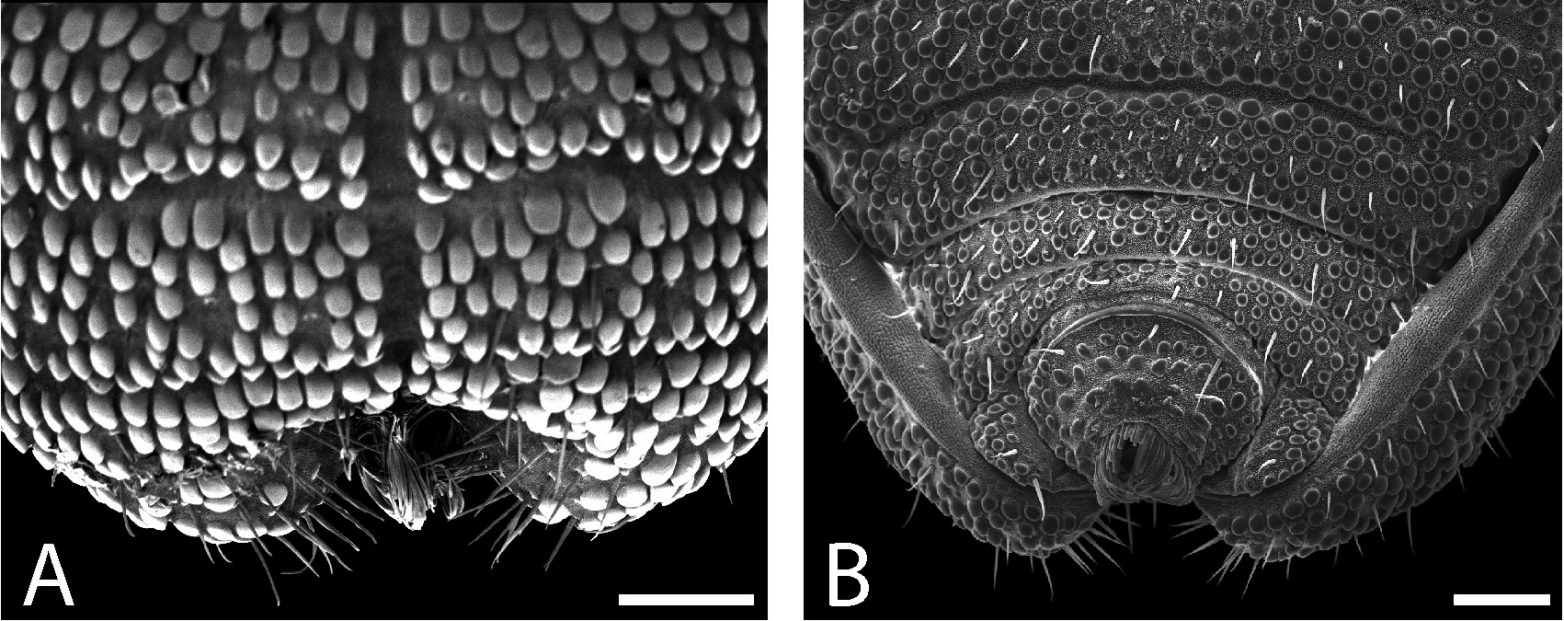
*Austropurcellia finniganensis* sp. n., paratype male, QM 102447. **A** dorsal view of posterior tergites **B** anal plate. Scale bars: 100 μm.

**Figure 9.**
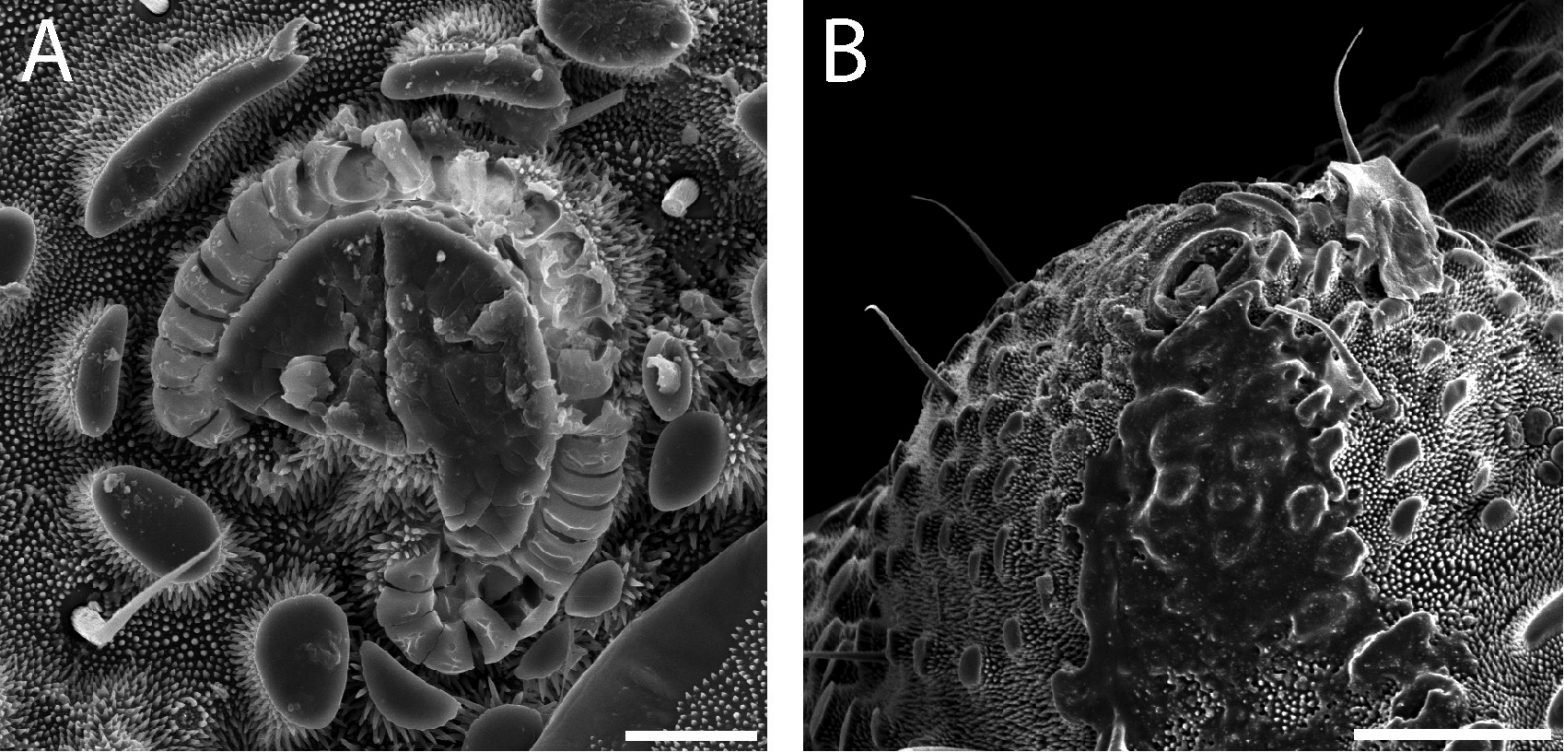
*Austropurcellia finniganensis* sp. n., males. **A** spiracle, QM 102447, paratype **B** ozophore, QM berlesate 981. Scale bar: 20 μm (**A**); 50 μm (**B**).

**Figure 10.**
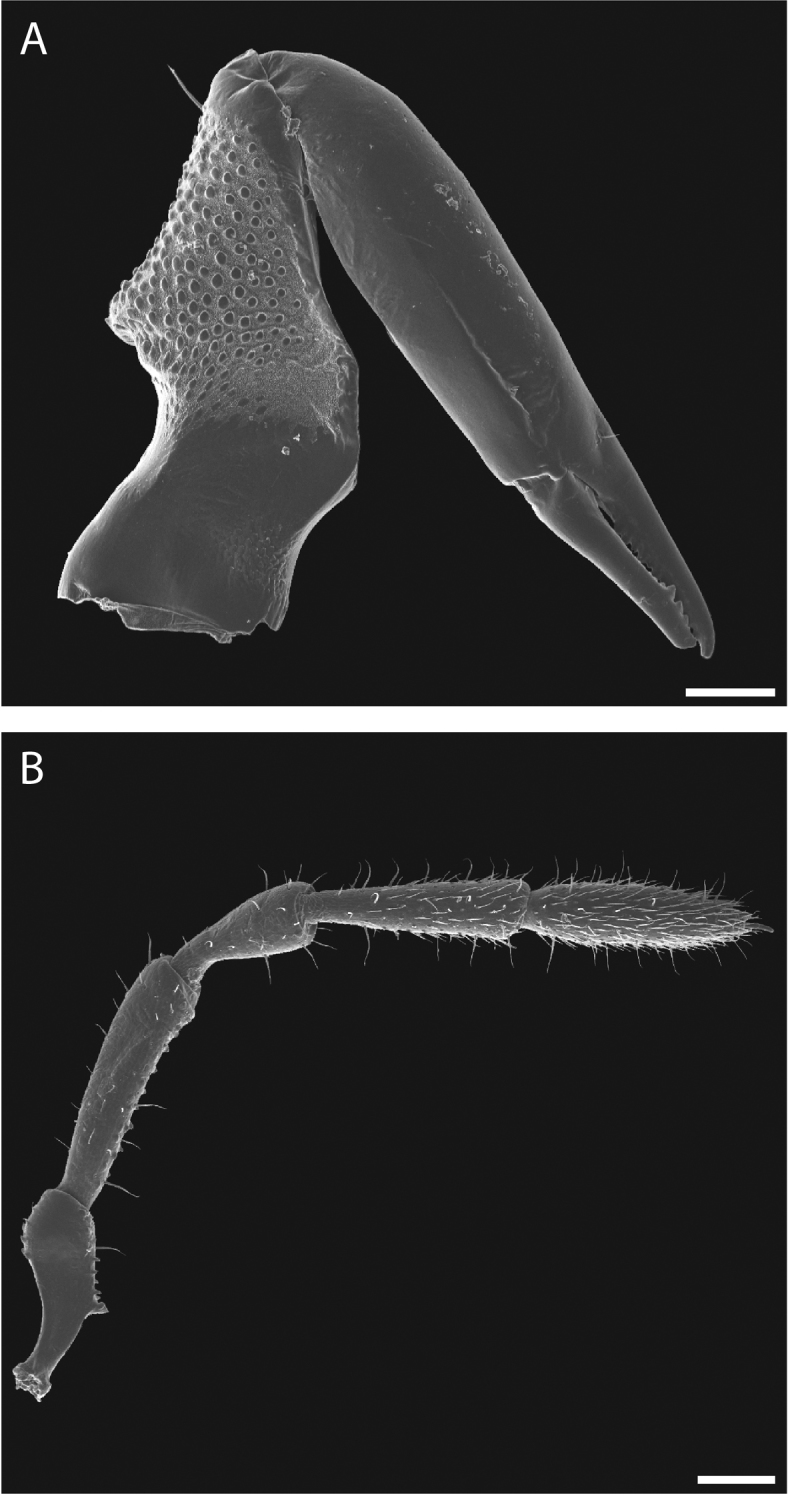
*Austropurcellia finniganensis* sp. n., paratype male and female, QM 102447. **A** female chelicera **B** male palp. Scale bars: 100 μm.

**Figure 11.**
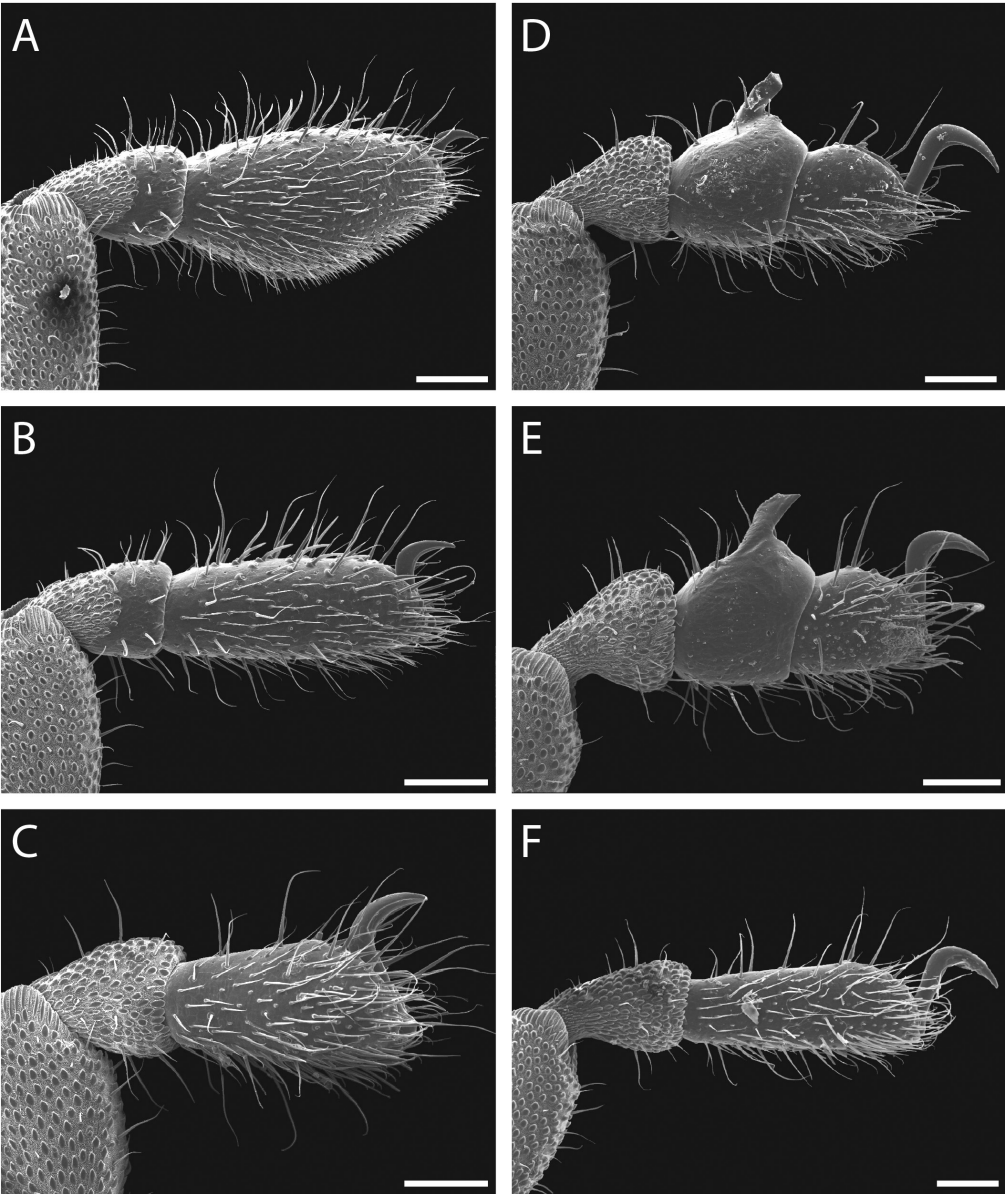
*Austropurcellia finniganensis* sp. n., paratype male and female, QM 102447. **A** male tarsus and metatarsus I **B** male tarsus and metatarsus II **C** female tarsus and metatarsus III **D** male tarsus and metatarsus IV, lateral view; **E** male tarsus and metatarsus IV, medial view **F** female tarsus and metatarsus IV. Scale bars: 100 μm.

Figs 6 , 7 , 8 , 9 , 10 , 11

##### Material examined.

*Holotype*. Male (QM 102446), Mt. Finnigan (sample 1, AR4), 15.816°S, 145.280°E, coll. Alex Riedel 28.iv.2014.

*Paratypes*. 3 males, 3 females, same collecting data as holotype, QM 102447, Macalester SEM stubs M30.11, M30.12.

##### Additional material.

1 female, Mt. Finnigan (sample 2, AR3), 15.816°S, 145.278°E, coll. Alex Riedel 28.iv.2014, MCZ IZ 68947.

4 males, 5 females, 2 juveniles, Mt. Finnigan 37 km S Cooktown, 15.817°S, 145.283°E, coll. G. B. Monteith, D. Yeates, and D. Cook 22.iv.1982, QM berlesate 401A, Macalester SEM stubs M8.1, M8.2.

3 males, 1 female, Mt. Finnigan Summit, 15.817°S, 145.283°E, coll. G. B. Monteith 21.xi.1998, QM berlesate 981, Macalester SEM stubs M6.11, M6.12.

##### Diagnosis.

Distinguished from congeners by very short, round scopula emerging from posterior quarter of fully granulated anal plate. Distinctive lack of granulation on sutures of dorsal scutum, including medial sulcus.

##### Description.

Pettalid with tergite VIII bilobed (Figs 6A–B, 7). Posterior margin of dorsal scutum curves ventrally (Fig. 6C). Length of male holotype (Fig. 6) 2.1 mm, width at widest point in posterior third of prosoma 1.2 mm, width at ozophores 0.8 mm. Most of body surface covered in microstructure of tubercles and granules (Fig. 7). Dorsal transverse sulci present and very prominent by lack of granulation (Fig. 7A). Dorsal longitudinal sulcus lacking granulation but with adjacent band of elongated granules oriented parallel to medial sulcus (Figs 7A, 8A). Granulation medially absent in anterior portions of sternites II-V; area of absent granulation approximately equal to width of gonostome (Fig. 7).

Ozophores relatively conical, of type III *sensu* Juberthie (1970) (Figs 7A, 9A). Coxae of legs I and II mobile, coxae of remaining legs fixed. Male coxae II–IV meeting in the midline (Fig. 7B). Male gonostome small, subtriangular, wider than long (Fig. 7B). Spiracles circular and C-shaped with slightly recurved edges (Fig. 9), as found in “open circle” type of Giribet and Boyer (2002). Anal region of “pettalid type” (Giribet and Boyer 2002). Anal plate convex and granulated (Fig. 8B). Short, round scopula extruding from posterior third of anal plate and extending just past posterior margin of anal plate (Fig. 8B). Orientation of scopula obscures anal pores, which are not visible (Fig. 8B).

Chelicerae (Fig. 10A) short and relatively robust. Proximal article of chelicerae with dorsal crest, without ventral process. Median article with prominent apodeme. Chela with two types of dentition typical in pettalids (Fig. 10A). Measurements of cheliceral articles of male paratype, from proximal to distal (in mm): 0.85, 0.94, 0.27. Palp (Fig. 10B) with prominent ventral process on trochanter. Measurements of palpal articles of male paratype from proximal to distal (in mm): 0.27, 0.28, 0.20, 0.31, 0.27.

Legs with all claws smooth, without ventral dentition or lateral pegs (Fig. 11). All tarsi smooth (Fig. 11). Distinct solea present on ventral surface of tarsus I (Fig. 11A). Metatarsi I and II heavily ornamented on proximal half, with distal half smooth (Fig. 11A, B). Remaining metatarsi with full ornamentation (Fig. 11C–F). Male tarsus IV completely divided into two tarsomeres (Fig. 11D, E). Adenostyle with relatively robust, pointed claw, wider base, and small pore at apex on lateral (external) side (Fig. 11D). Seta on lateral surface of adenostyle (Fig. 11D–E) (example with adenostyle features labeled, Fig. 5).

Length measurements from male paratype of leg articles from proximal to distal (in mm): leg I [trochanter damaged], 0.59, 0.26, 0.43, 0.20, 0.40; leg II 0.26, 0.42, 0.25, 0.30, 0.15, 0.34; leg III [trochanter damaged], 0.48, 0.20, 0.33, 0.15, 0.31; leg IV [trochanter damaged], 0.50, 0.24, 0.39, 0.16, 0.35. Width measurements from male paratype of leg articles from proximal to distal (in mm): leg I [trochanter damaged], 0.18, 0.18, 0.17, 0.15, 0.20; leg II 0.17, 0.15, 0.16, 0.18, 0.13, 0.14; leg III [trochanter damaged], 0.18, 0.18, 0.19, 0.12, 0.14; leg IV 0.16, 0.21, 0.20, 0.21, 0.17, 0.18.

##### Etymology.

The specific epithet refers to the type locality, Mt. Finnigan.

#### Austropurcellia fragosa

Taxon classificationAnimaliaOpilionesPettalidae

Popkin-Hall, Jay & Boyer sp. n.

http://zoobank.org/9DE6E237-E3C7-411C-95D6-3441E83C22BB

**Figure 12.**
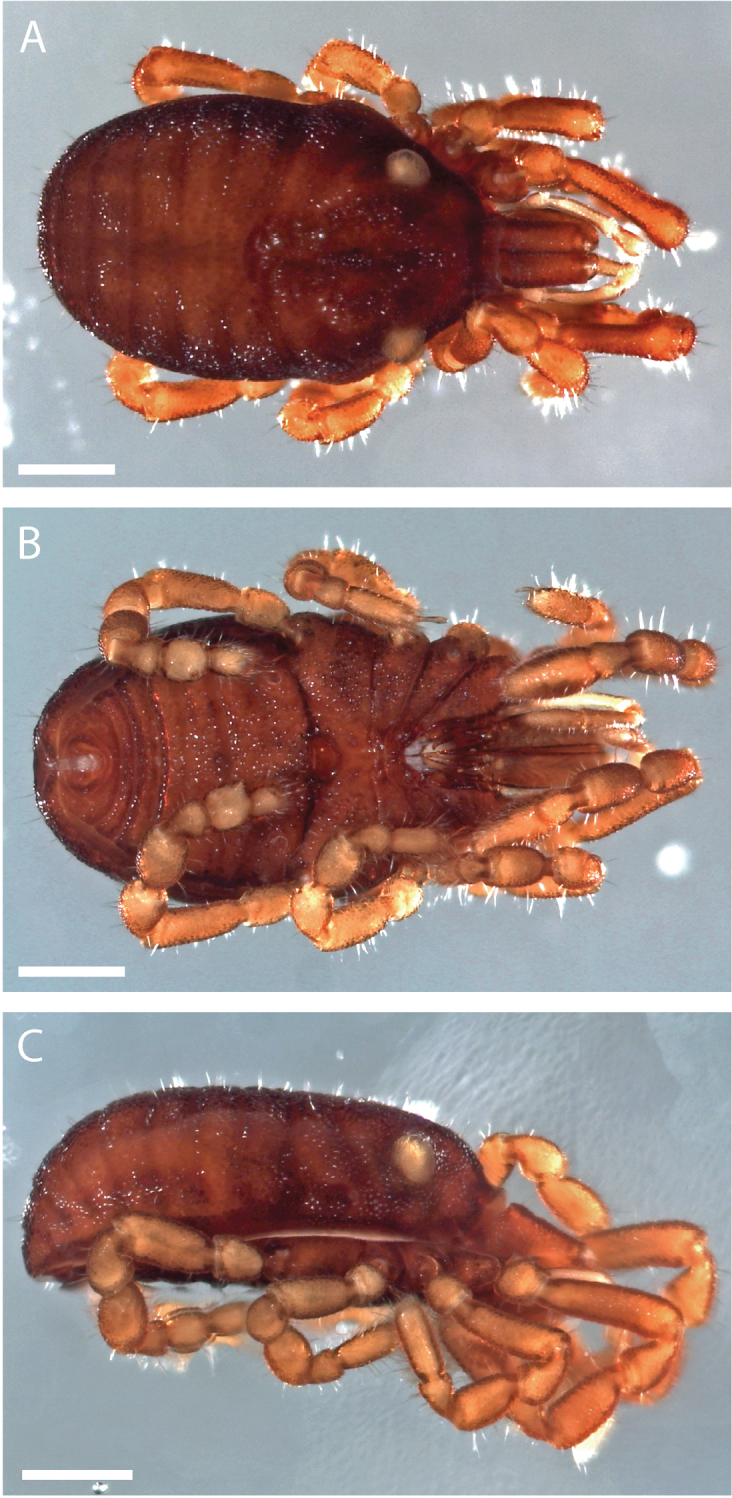
*Austropurcellia fragosa* sp. n., holotype male, QM 102445. **A** dorsal view **B** ventral view **C** lateral view. Scale bars: 0.5 mm.

**Figure 13.**
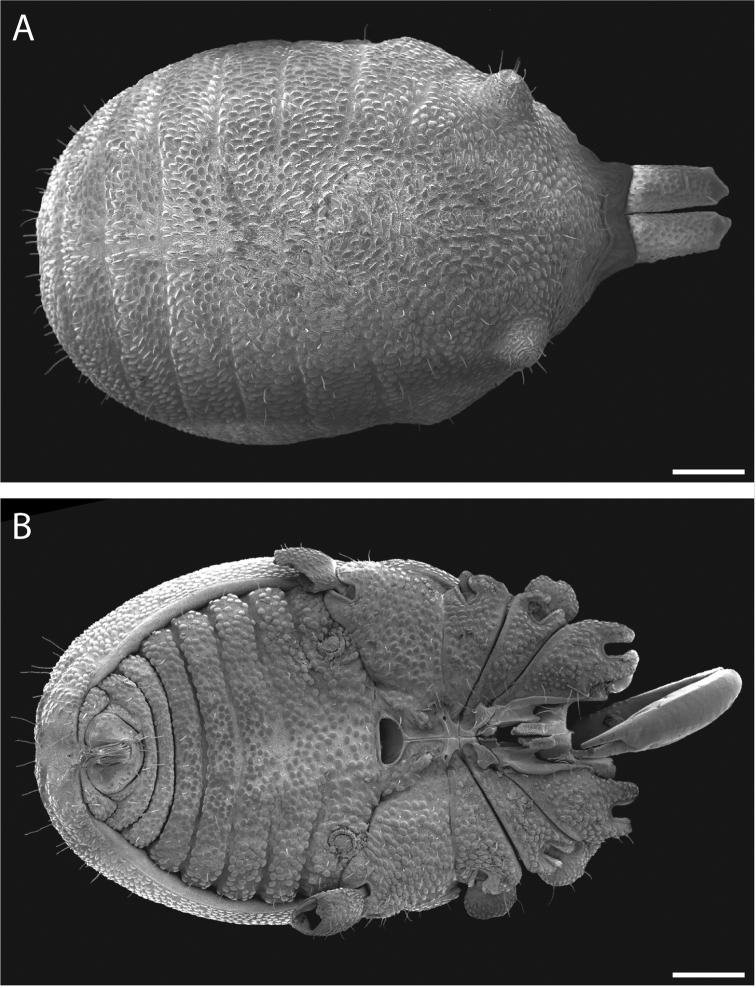
*Austropurcellia fragosa* sp. n., males. **A** dorsal view, QM berlesate 38121, paratype **B** ventral view, QM berlesate 448. Scale bars: 200 μm.

**Figure 14.**
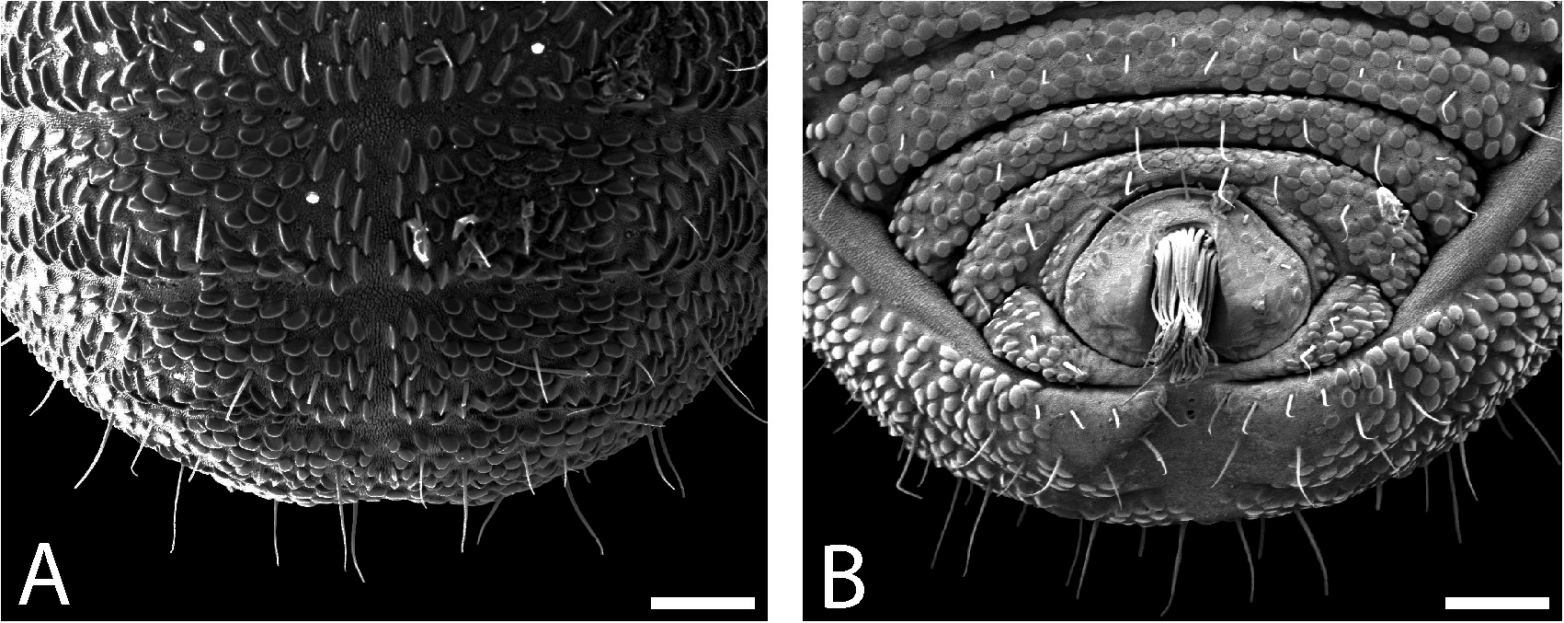
*Austropurcellia fragosa* sp. n., males. **A** dorsal view of posterior tergites, QM berlesate 453 **B** anal plate, QM berlesate 448. Scale bars: 100 μm.

**Figure 15.**
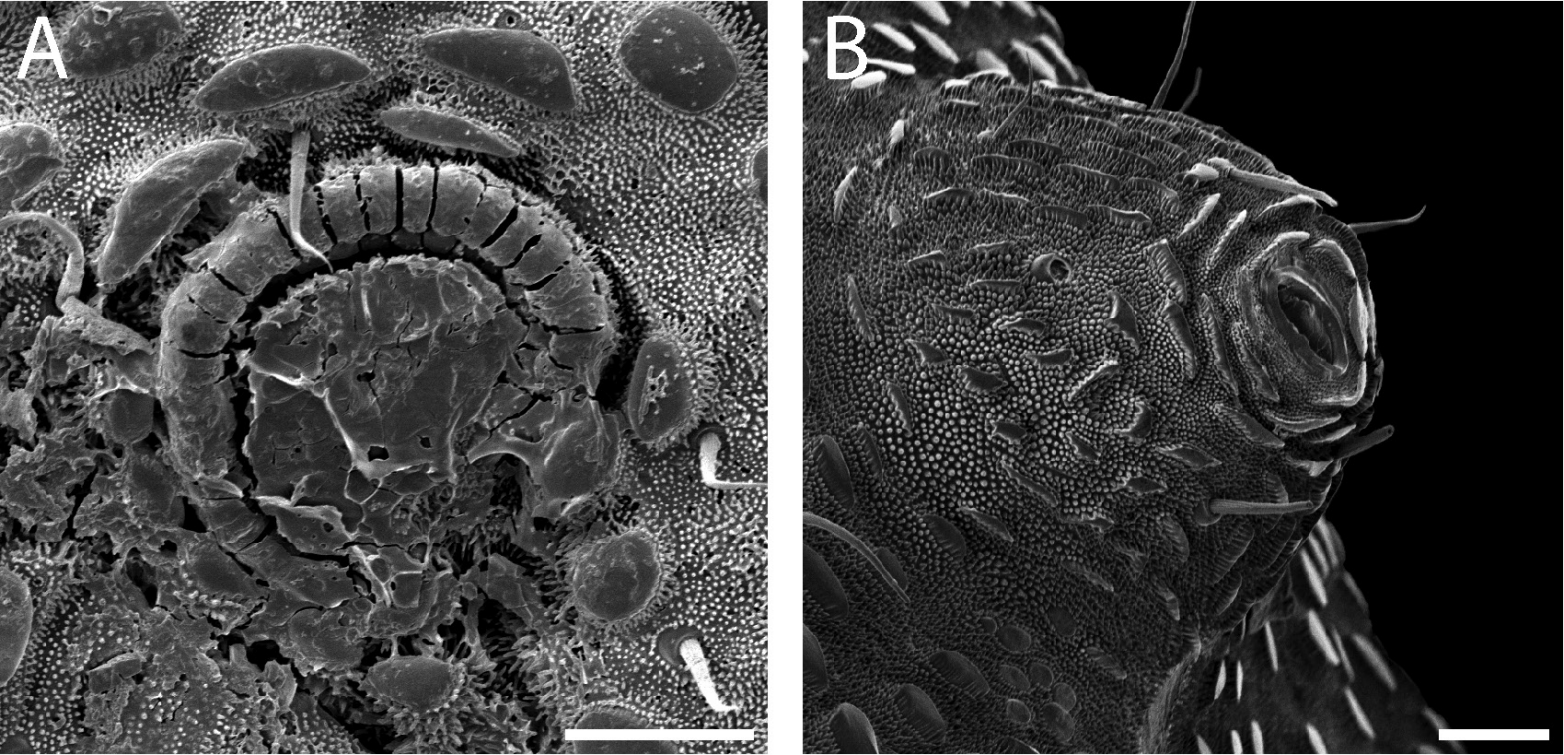
*Austropurcellia fragosa* sp. n., males. **A** spiracle, QM berlesate 448 **B** ozophore, QM berlesate 453. Scale bar: 20 μm (**A**); 50 μm (**B**).

**Figure 16.**
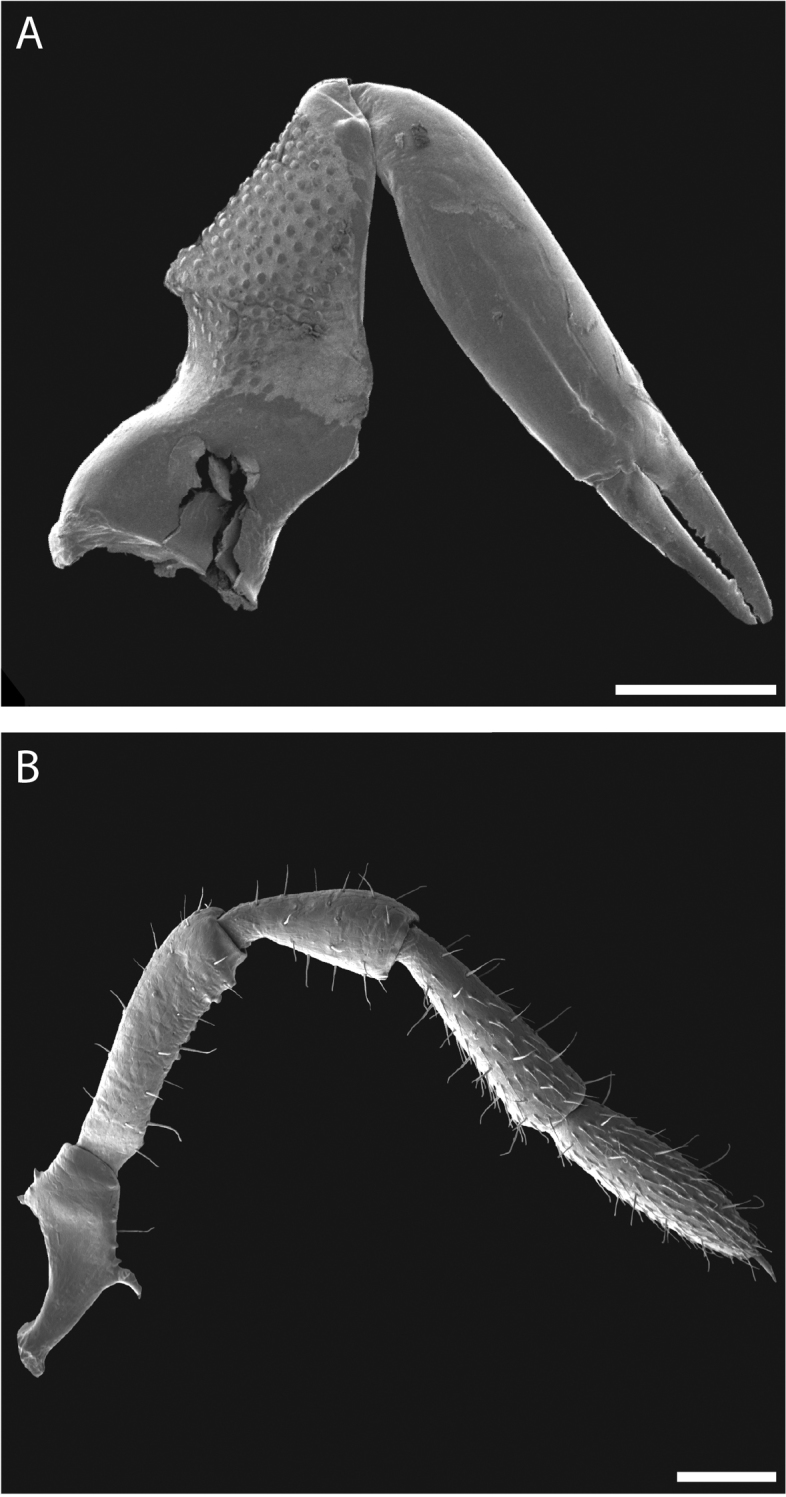
*Austropurcellia fragosa* sp. n., males. **A** chelicera, QM berlesate 448 **B** palp, QM berlesate 453. Scale bar: 200 μm (**A**); 100 μm (**B**).

**Figure 17.**
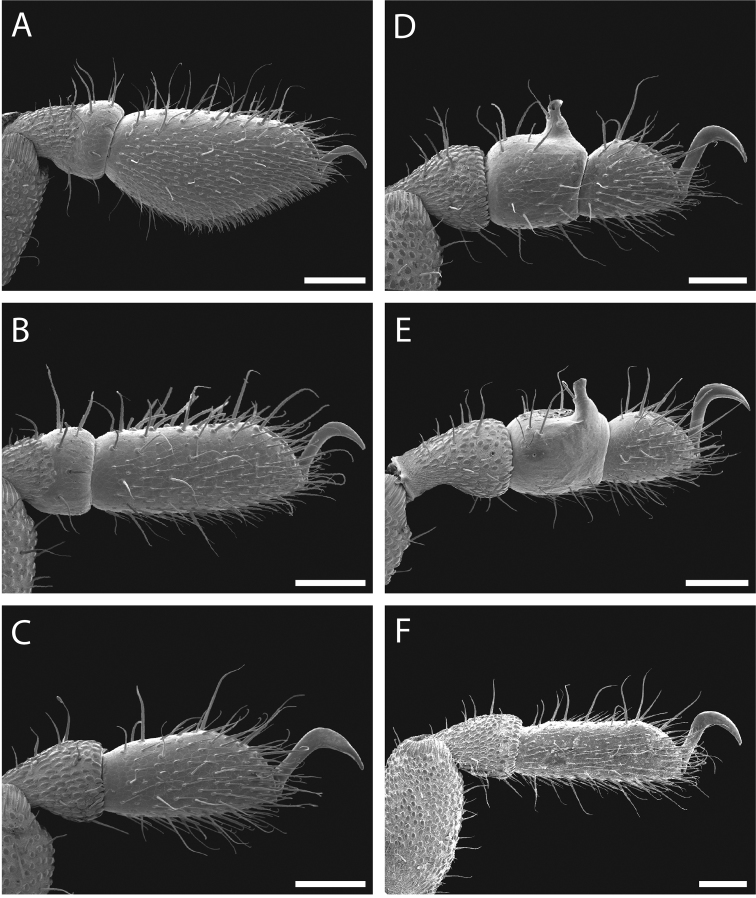
*Austropurcellia fragosa* sp. n., males and female. **A** male tarsus and metatarsus I, QM berlesate 448 **B** male tarsus and metatarsus II, QM berlesate 448; **C** male tarsus and metatarsus III, QM berlesate 448 **D** male tarsus and metatarsus IV, lateral view, QM berlesate 448 **E** male tarsus and metatarsus IV, medial view, QM berlesate 453 **F** female tarsus and metatarsus IV, QM berlesate 684. Scale bars: 100 μm.

Figs 12 , 13 , 14 , 15 , 16 , 17

##### Material examined.

*Holotype*. Male (QM 102445 [ex QM 38121]), Roaring Meg Creek, 16.074°S, 145.416°E, coll. K. Aland and G. B. Monteith 1.v.2015, QM 38121.

*Paratypes*. 5 males, 2 females, same collecting data as holotype, QM 38121, Macalester SEM stub M30.10.

##### Additional material.

2 females, 4 juveniles, McDowall Range 17 km N Daintree, 16.100°S, 145.333°E, coll. G. B. Monteith 27.xi.1985, QM berlesate 684.

1 male, 2 females, Roaring Meg Creek 6 km W Cape Tribulation, 16.083°S, 145.4°E, coll. G. B. Monteith, D. Yeates, G. Thompson 5.x.1982, QM berlesate 448, Macalester SEM stubs M22.11, M22.12.

1 female, Roaring Meg Creek 6 km W Cape Tribulation, 16.067°S, 145.400°E, coll. G. B. Monteith, D. Yeates, G. Thompson 5.x.1982, QM berlesate 453, Macalester SEM stubs M22.9, M22.10.

##### Diagnosis.

Distinguished from congeners by convex anal plate with long, narrow scopula emerging from anterior quarter of anal plate and occupying a rectangular indented area for its entire length. Distinctive ungranulated areas cause ventral sutures to appear fused.

##### Description.

Pettalid with tergite VIII bilobed (Fig. 13). Length of male holotype (Fig. 12) 2.0 mm, width at widest point in posterior third of prosoma 1.2 mm, width at ozophores 0.8 mm. Most of body surface covered in microstructure of tubercles and granules (Fig. 13). Posterior ventral body margin flexed anteriorly. Dorsal transverse sulci present and distinct by lack of granulation (Figs 13A, 14A). Dorsal longitudinal sulcus lacking granulation, with adjacent band of elongated granules flanking dorsal longitudinal sulcus (Figs 13A, 14A). Granulation medially absent in anterior portions of sternites II-VI; area of absent granulation approximately equal to width of gonostome (Fig. 13B).

Ozophores relatively tall and conical, of type III *sensu* Juberthie (1970) (Figs 13A, 15B). Coxae of legs I and II mobile, coxae of remaining legs fixed. Male coxae II–IV meeting in the midline (Fig. 13B). Male gonostome small, subtriangular, wider than long (Fig. 13B). Spiracles circular and C-shaped with slightly recurved edges (Fig. 15A), as found in “open circle” type of Giribet and Boyer (2002). Anal region of “pettalid type” (Giribet and Boyer 2002). Anal plate convex and largely ungranulated, with light granulation along anterior margin (Fig. 14B). Long, narrow scopula emerging at anterior quarter of anal plate and extending past posterior margin of anal plate (Fig. 14B). Scopula inset into rectangular area at center of anal plate (Fig. 14B). Three anal pores visible, located between lobes of tergite VIII (Fig. 14B).

Chelicerae (Fig. 16A) short and relatively robust. Proximal article of chelicerae with dorsal crest, without ventral process. Median article with prominent apodeme. Chela with two types of dentition typical in pettalids (Fig. 16A). Measurements of cheliceral articles of male paratype from proximal to distal (in mm): 0.70, 0.76, 0.24. Palp (Fig. 16B) with prominent ventral process on trochanter. Measurements of palpal articles of male paratype from proximal to distal (in mm): 0.22, 0.27, 0.15, 0.22, 0.23.

Legs with all claws smooth, without ventral dentition or lateral pegs (Fig. 17). All tarsi smooth (Fig. 17). Distinct solea present on ventral surface of tarsus I (Fig. 17A). Metatarsi I and II heavily ornamented on proximal half, with smooth distal half (Fig. 17A, B). Remaining metatarsi with full ornamentation (Fig. 17C–F). Male tarsus IV completely divided into two tarsomeres (Fig. 17D, E). Adenostyle with relatively robust, blunt claw, wide base, and small pore at apex on lateral (external) side (Fig. 17D). Long seta on lateral surface of adenostyle from below pore to above apex (Fig. 17D, E); very short seta rising from adenostyle base below pore (Fig. 17D) (example with adenostyle features labeled, Fig. 5).

Measurements from male paratype of leg articles from proximal to distal (in mm): leg I [trochanter damaged], 0.51, 0.17, 0.33, 0.18, 0.35; leg II [trochanter damaged], 0.38, 0.18, 0.27, 0.14, 0.30; leg III 0.14, 0.29, 0.18, 0.24, 0.12, 0.26; leg IV [trochanter damaged], 0.42, 0.23, 0.29, 0.17, 0.31. Width measurements from male paratype of leg articles from proximal to distal (in mm): leg I [trochanter damaged], 0.16, 0.16, 0.16, 0.14, 0.20; leg II [trochanter damaged], 0.15, 0.15, 0.17, 0.12, 0.12; leg III 0.16, 0.16, 0.15, 0.17, 0.12, 0.13; leg IV [trochanter damaged], 0.18, 0.17, 0.19, 0.14, 0.15.

##### Etymology.

The specific epithet is derived from the first declension form of *fragōsus*, from Latin, meaning “roaring” or “crashing”, a reference to the type locality, Roaring Meg Creek.

#### Austropurcellia megatanka

Taxon classificationAnimaliaOpilionesPettalidae

Jay, Coblens & Boyer sp. n.

http://zoobank.org/16C62C3B-BCE8-4EC0-8CD5-14D81C68ED0F

**Figure 18.**
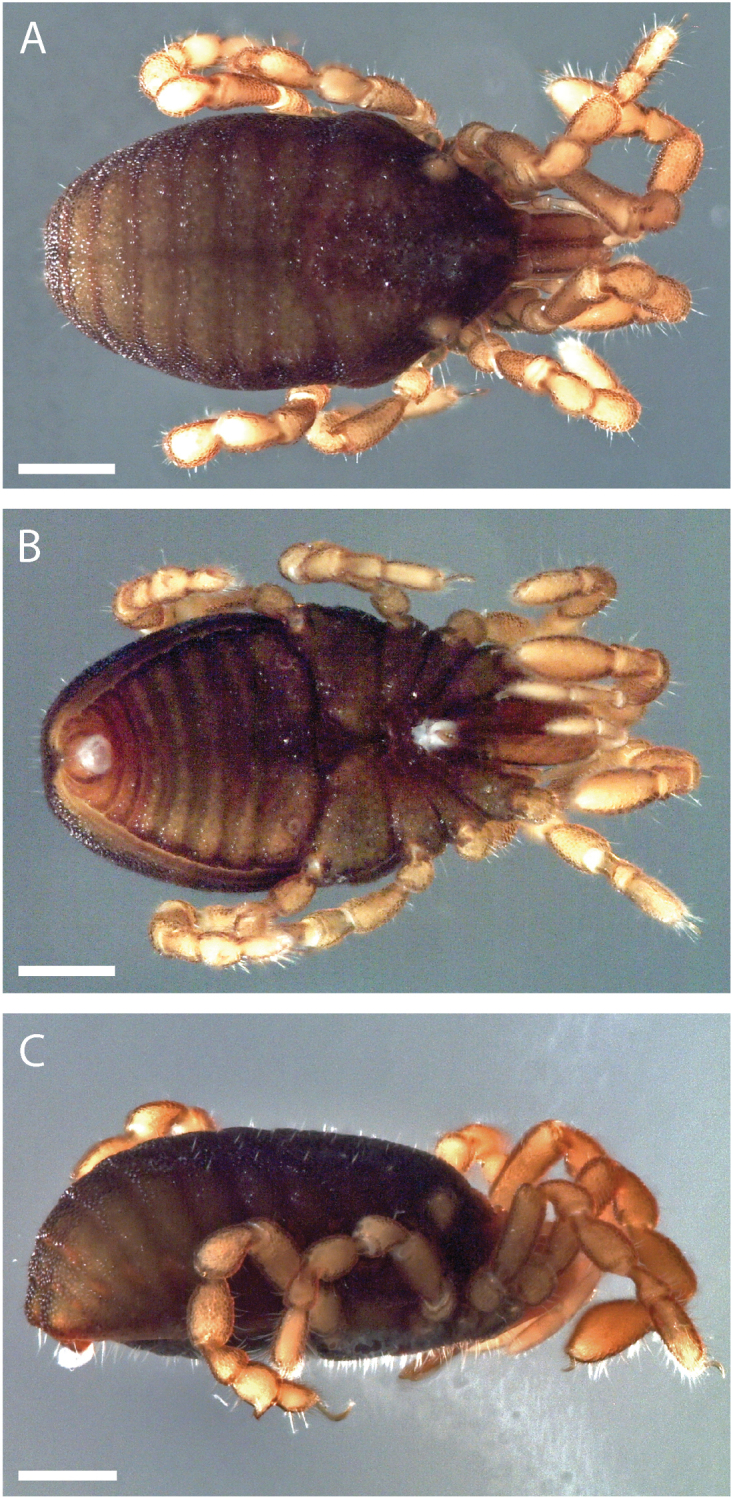
*Austropurcellia megatanka* sp. n., holotype male, QM 102440. **A** dorsal view **B** ventral view **C** lateral view. Scale bars: 0.5 mm.

**Figure 19.**
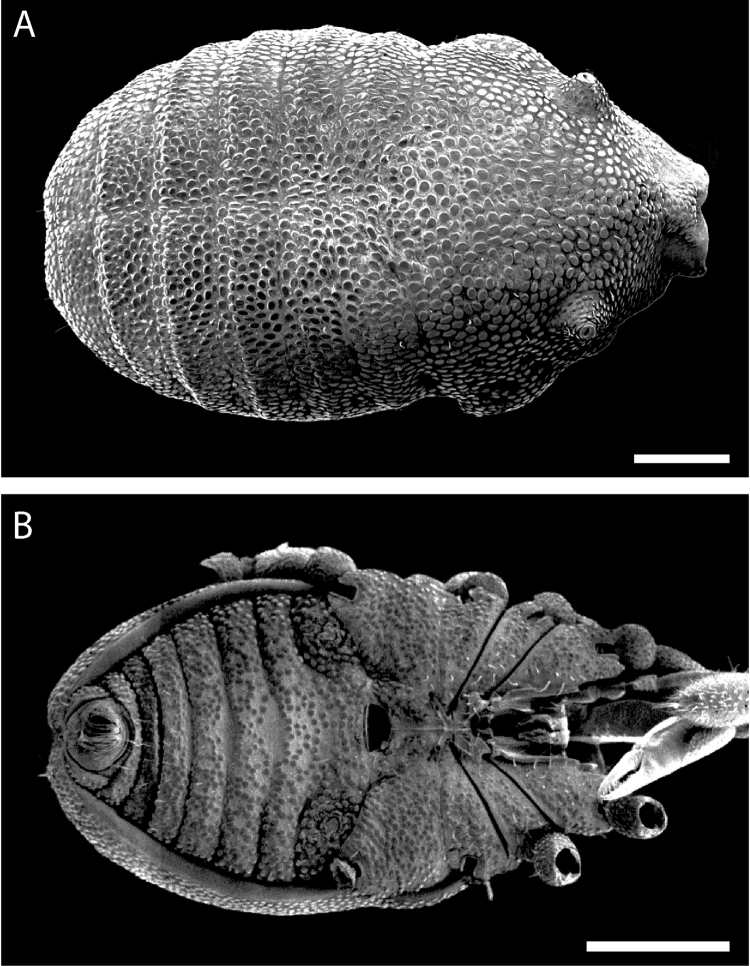
*Austropurcellia megatanka* sp. n., paratype male, QM 102441. **A** dorsal view **B** ventral view. Scale bar: 200 μm (**A**); 500 μm (**B**).

**Figure 20.**
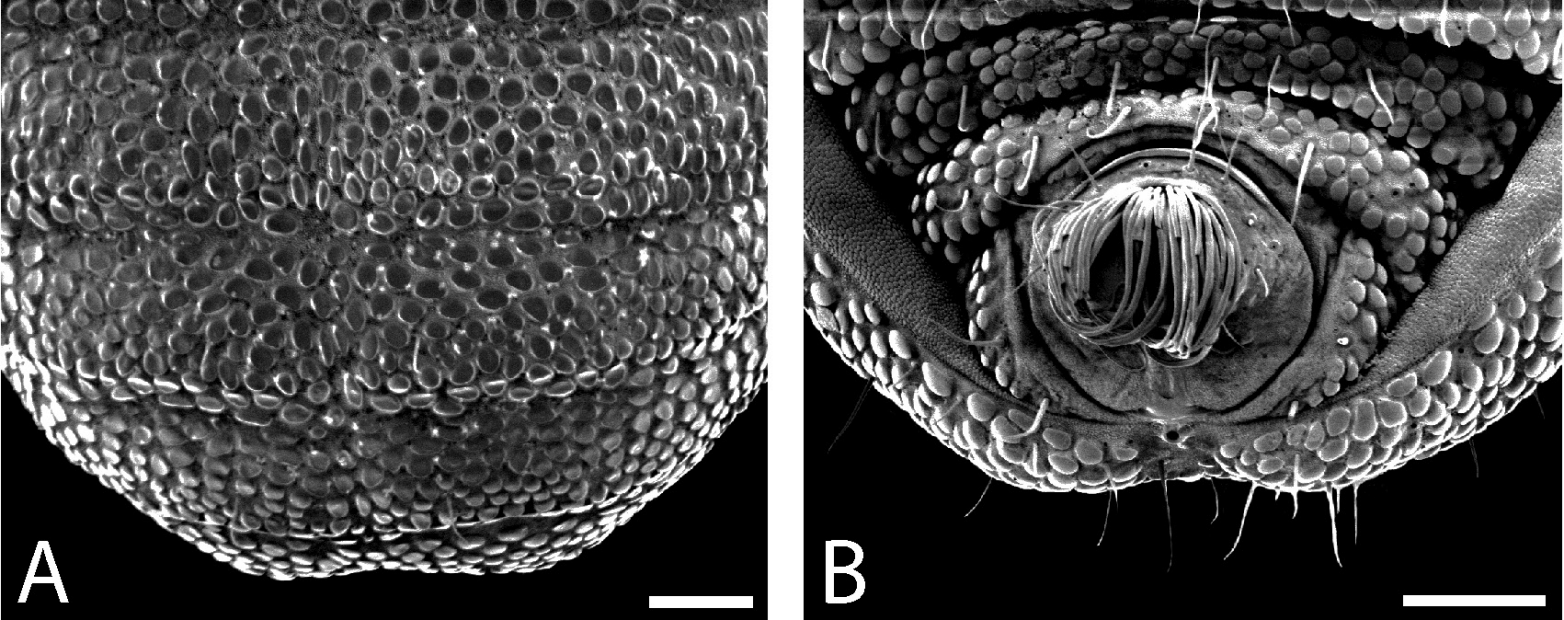
*Austropurcellia megatanka* sp. n., paratype male, QM 102441. **A** dorsal view of posterior tergites **B** anal plate. Scale bars: 100 μm.

**Figure 21.**
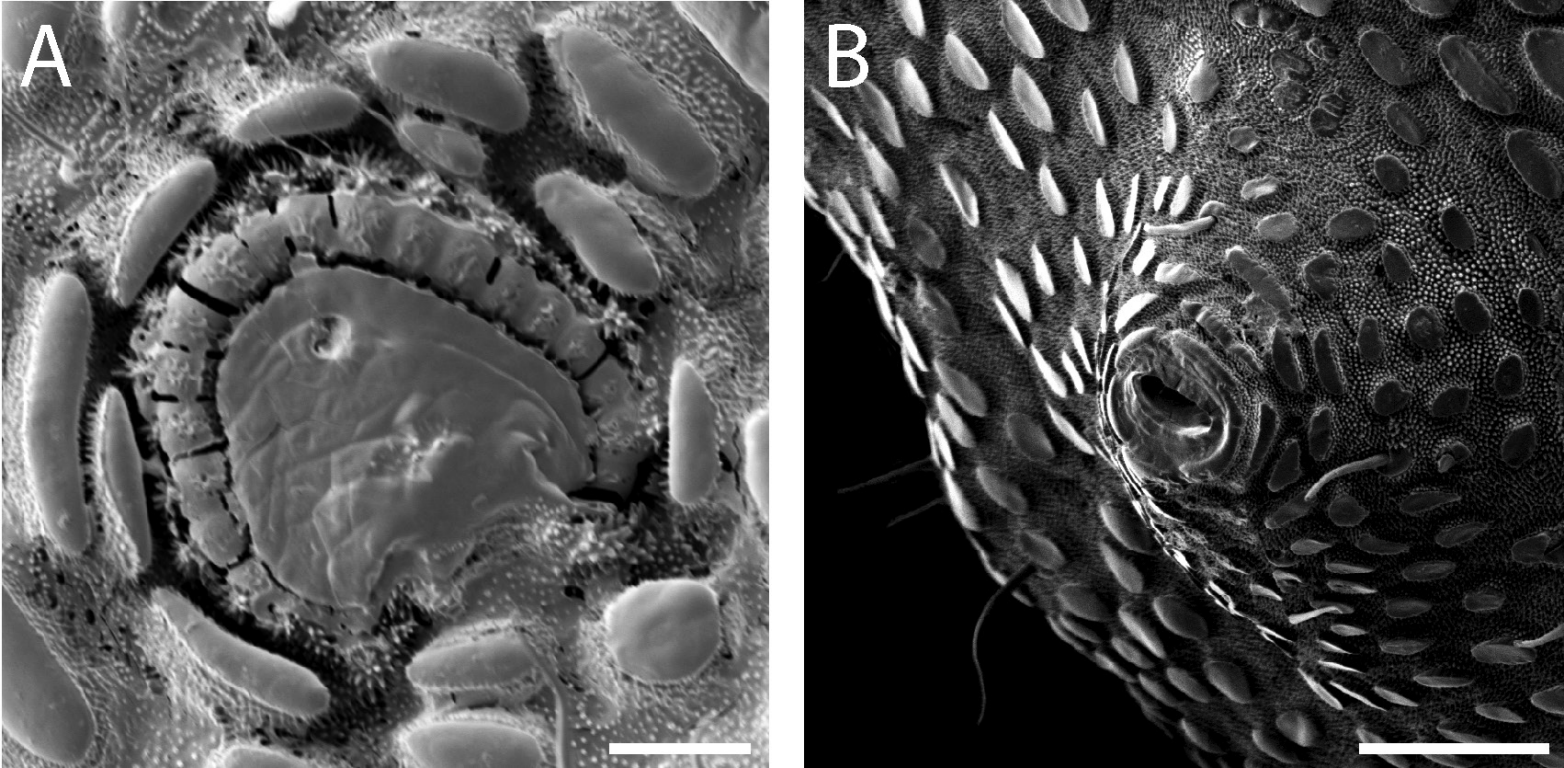
*Austropurcellia megatanka* sp. n., males. **A** spiracle, QM berlesate 918 **B** ozophore, QM 102441, paratype. Scale bar: 20 μm (**A**); 50 μm (**B**).

**Figure 22.**
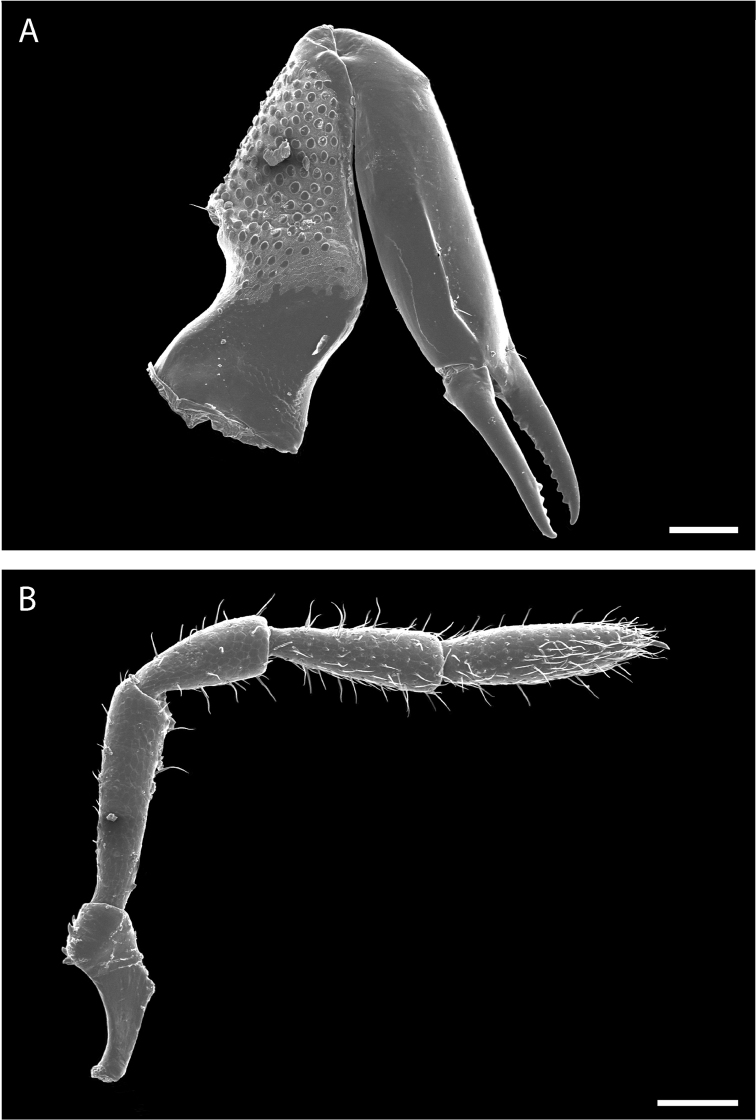
*Austropurcellia megatanka* sp. n., paratype male, QM 102441. **A** chelicera **B** palp. Scale bars: 100 μm.

**Figure 23.**
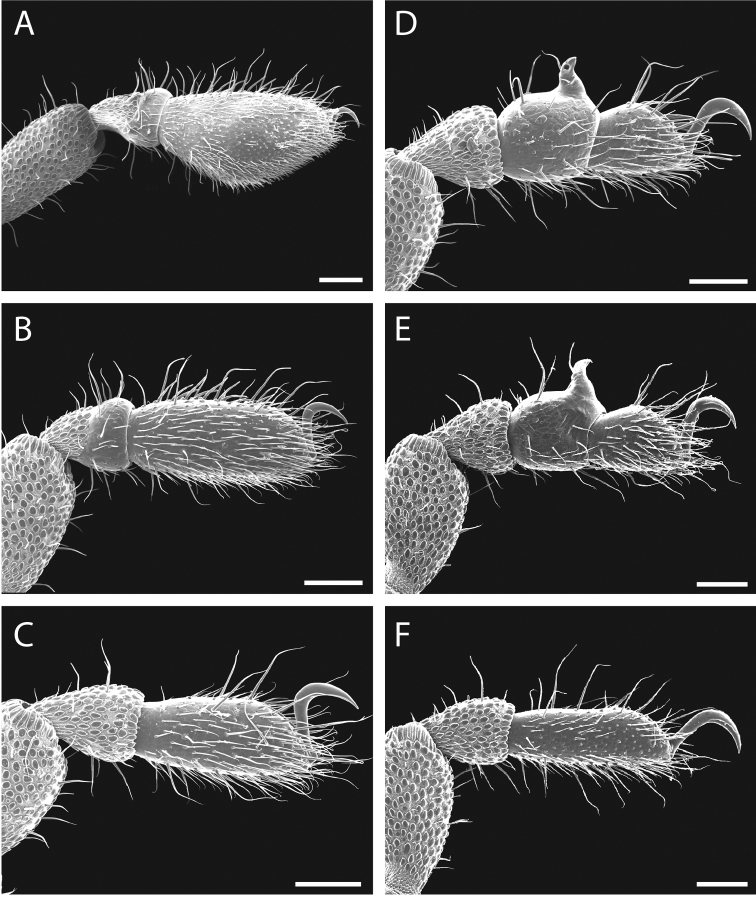
*Austropurcellia megatanka* sp. n., paratype male and female, QM 102441. **A** male tarsus and metatarsus I **B** male tarsus and metatarsus II; **C** male tarsus and metatarsus III **D** male tarsus and metatarsus IV, lateral view **E** male tarsus and metatarsus IV, medial view **F** female tarsus and metatarsus IV. Scale bars: 100 μm.

Figs 18 , 19 , 20 , 21 , 22 , 23

##### Material examined.

*Holotype*. Male (QM 102440 [ex MCZ IZ 68951]), Baldy Mountain Road, Herberton Range National Park, 17.287°S, 145.427°E, coll. S. L. Boyer, M. J. Coblens, K. R. Jay and P. P. Sharma 29.v.2014.

*Paratypes*. 2 males, 1 female, QM 102441 (ex MCZ IZ 68948), same collecting data as holotype. 2 males, 1 female, 3 juveniles, same collecting data as holotype, MCZ IZ 68949, Macalester SEM stubs M27.7, M27.8, M30.2.

##### Additional material.

1 male, 3 females, Baldy Mountain Road, Herberton Range National Park, 17.267°S, 145.267°E, coll. D. Yeates and D. Cook 25.xi.1985. QM berlesate 683, S 1755, Macalester SEM stubs M23.3, M23.4.

4 males, 1 female, Baldy Mountain, 17.284°S, 145.432°E, coll. G. B. Monteith 10.x.1980, QM S 2281.

1 male, CSIRO Trail (after hut), 17.108°S, 145.629°E, coll. S. L. Boyer, M. J. Coblens, K. R. Jay and P. P. Sharma 29.v.2014, MCZ IZ 68950.

4 juveniles, Mt. Haig, 17.1°S, 145.583°E, coll. Taylor and Feehan 30.vi.1971, ANIC 349.

1 male, 3 females, 5 juveniles, Mt. Haig, Lamb Range, 17.083°S, 145.6°E, coll. G. B. Monteith 25.ii.1997, QM berlesate 918, Macalester SEM stubs M20.11, M20.12.

4 males, 2 females, 22 juveniles, Mt. Tiptree, 17.067°S, 145.617°E, coll. Taylor and Feehan 29.vi.1971, ANIC 345, ANIC 346, ANIC 347, ANIC 348.

##### Diagnosis.

Distinguished from congeners by an usually wide and long scopula emerging from anterior quarter of male anal plate and easily visible in lateral view. Anal plate is very flat compared to the more rounded anal plates of geographically proximate species such as *Austropurcellia tholei* and *Austropurcellia despectata*. Distinctive areas lacking granulation cause ventral sutures to appear fused. Male tarsus IV is fully bisegmented rather than partially bisegmented as in *Austropurcellia tholei* and *Austropurcellia despectata*.

##### Description.

Pettalid with tergite VIII bilobed (Fig. 19). Length of male holotype (Fig. 18) 2.1 mm, width at widest point in posterior third of prosoma 1.2 mm, width at ozophores 0.8 mm. Most of body surface covered in microstructure of tubercles and granules (Fig. 19). Transverse sulci present and granulated (Fig. 19A). Dorsal longitudinal sulcus granulose (Figs 19A, 20A). Granulation medially absent in sternites II-V; area of absent granulation approximately equal to width of gonostome (Fig. 19B).

Ozophores relatively conical, of type III *sensu* Juberthie (1970) (Figs 19A, 21B). Coxae of legs I and II mobile, coxae of remaining legs fixed. Male coxae II–IV meeting in the midline (Fig. 19B). Male gonostome small, subtriangular, and wider than long (Fig. 19). Spiracles circular and C-shaped with slightly recurved edges, as found in “open circle” type of Giribet and Boyer (2002) (Fig. 21). Anal region of “pettalid type” (Giribet and Boyer 2002). Anal plate flat and largely ungranulated (Fig. 20B). Long, full scopula emerging from anterior quarter of anal plate and curling into toward posterior quarter of anal plate (Fig. 20B). Anal pore visible (Fig. 20B).

Chelicerae (Fig. 22A) short and relatively robust. Proximal article of chelicerae with dorsal crest, without ventral process. Median article with prominent apodeme. Chela with two types of dentition typical in pettalids (Fig. 22A). Measurements of cheliceral articles of male paratype from proximal to distal (in mm): 0.51, 0.72, 0.25. Palp (Fig. 22B) with prominent ventral process on trochanter. Measurements of palpal articles of male paratype from proximal to distal (in mm): 0.20, 0.25, 0.17, 0.22, 0.24.

Legs with all claws smooth, without ventral dentition or lateral pegs (Fig. 23). All tarsi smooth (Fig. 23). Distinct solea present on ventral surface of tarsus I (Fig. 23A). Metatarsi I and II heavily ornamented on proximal half, with distal half smooth (Fig. 23A, B). Remaining metatarsi with full ornamentation (Fig. 23C–F). Male tarsus IV fully divided into two tarsomeres (Fig. 23D, E). Adenostyle with relatively robust claw, wide base, and small pore at apex on lateral (external) side (Fig. 23D). Long seta rising from medial (internal) face of adenostyle from below pore to above apex (Fig. 23D, E); very short seta rising from adenostyle base below pore on lateral (external) face (Fig. 23D) (example with adenostyle features labeled, Fig. 5).

Measurements from male paratype of leg articles from proximal to distal (in mm): leg I 0.14, 0.48, 0.20, 0.30, 0.16, 0.39; leg II [trochanter damaged], 0.37, 0.17, 0.26, 0.13, 0.32; leg III [trochanter damaged], 0.30, 0.18, 0.23, 0.15, 0.26; leg IV [trochanter damaged], 0.36, 0.20, 0.28, 0.14, 0.32. Width measurements from male paratype of leg articles from proximal to distal (in mm): leg I [trochanter damaged], 0.15, 0.17, 0.16, 0.14, 0.19; leg II [trochanter damaged], 0.14, 0.16, 0.17, 0.12, 0.13; leg III 0.18, 0.15, 0.15, 0.16, 0.12, 0.11; leg IV 0.16, 0.17, 0.16, 0.18, 0.14, 0.14.

##### Etymology.

The specific epithet, a noun in apposition, honors a Mitsubishi Pajero four-wheel drive vehicle, nicknamed ‘Big Tank,’ which made it possible to access various remote localities in the WT, including the type locality of *Austropurcellia megatanka* sp. n.

#### Austropurcellia monteithi

Taxon classificationAnimaliaOpilionesPettalidae

Jay, Popkin-Hall, Coblens & Boyer sp. n.

http://zoobank.org/BF2F8D6F-27F6-4421-BD92-43C6676602ED

**Figure 24.**
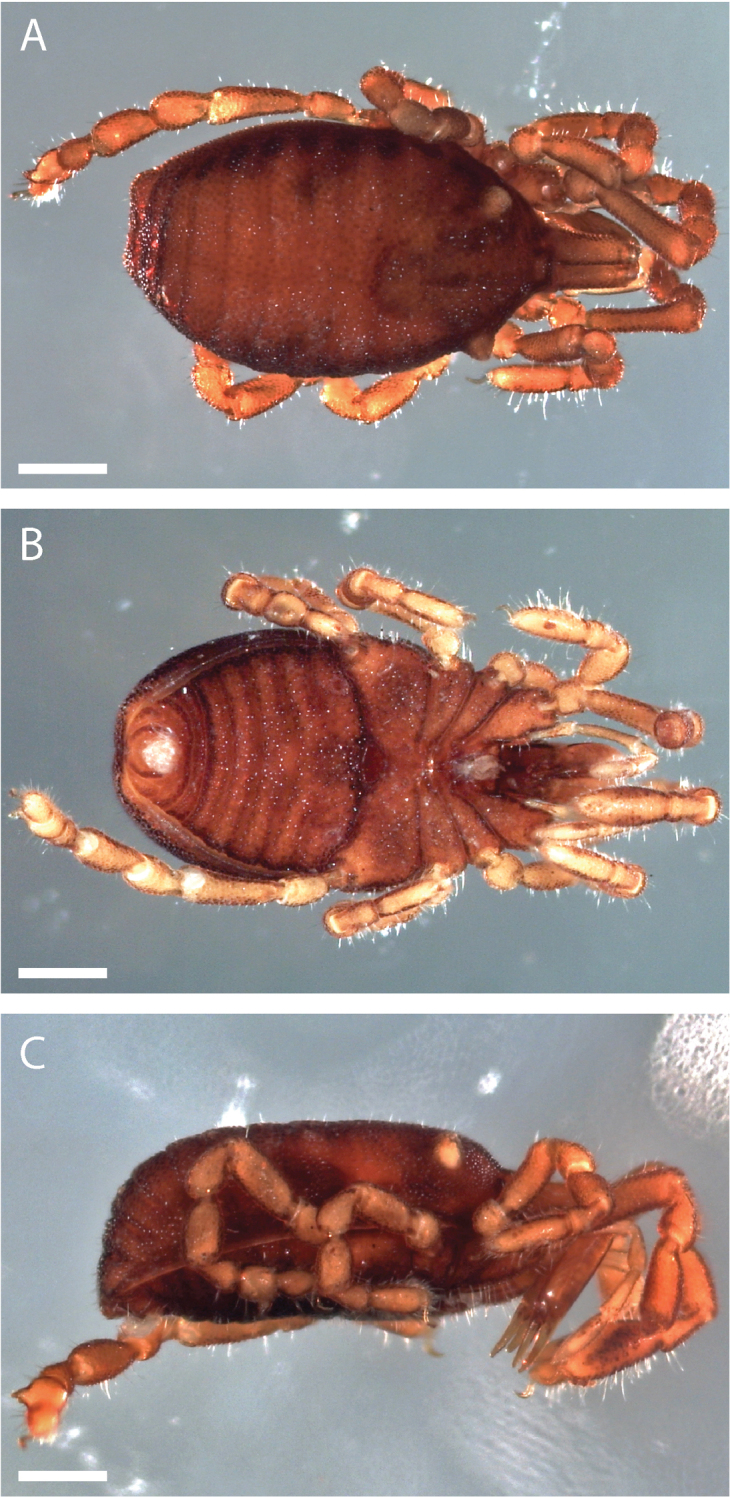
*Austropurcellia monteithi* sp. n., holotype male, QM 102442. **A** dorsal view **B** ventral view **C** lateral view. Scale bars: 0.5 mm.

**Figure 25.**
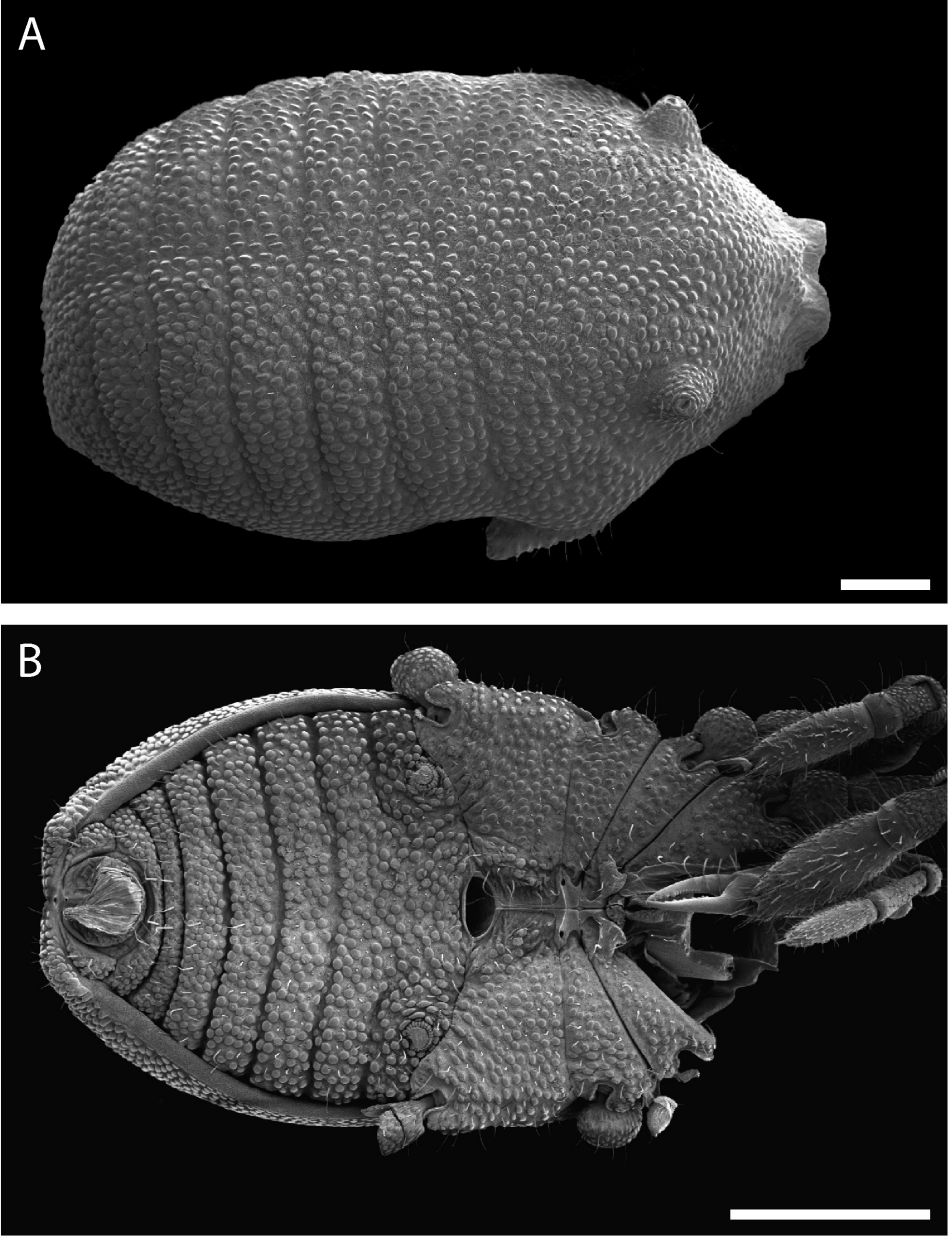
*Austropurcellia monteithi* sp. n., paratype male, QM 102443. **A** dorsal view **B** ventral view. Scale bar: 200 μm (**A**); 500 μm (**B**).

**Figure 26.**
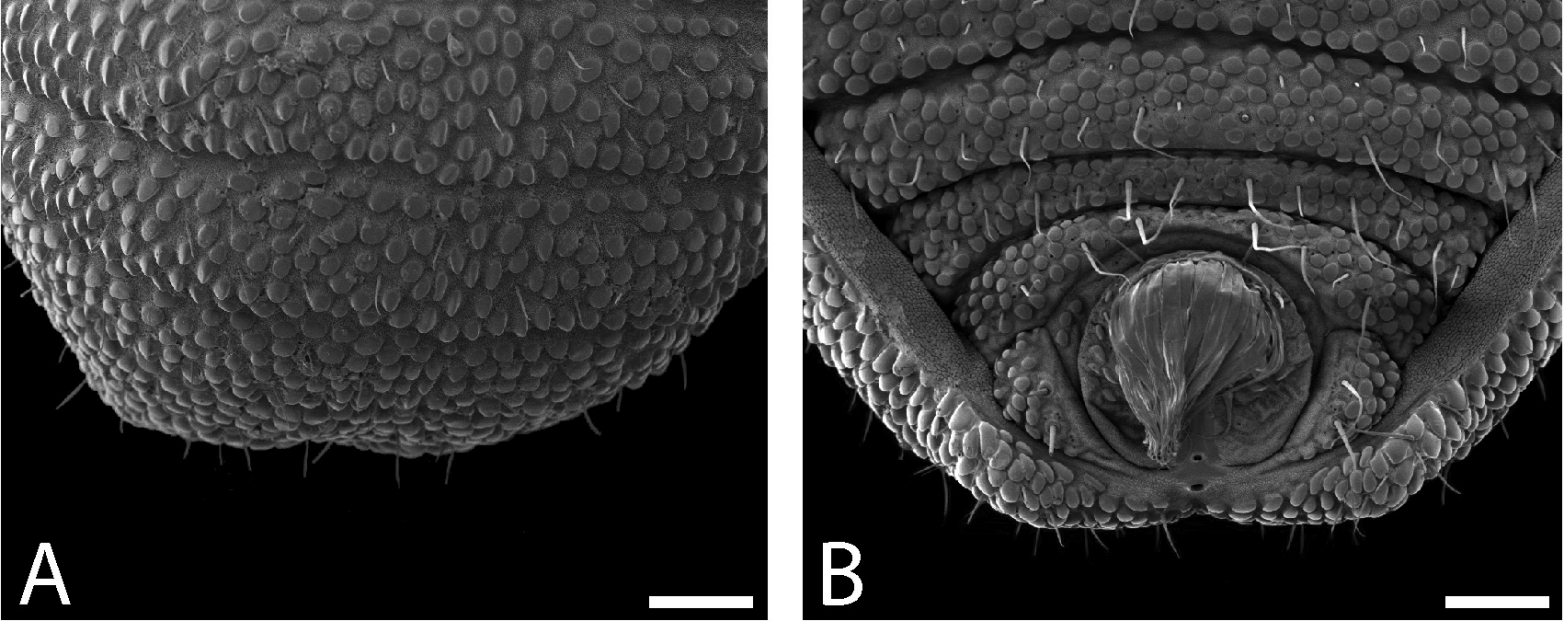
*Austropurcellia monteithi* sp. n., paratype male, QM 102443. **A** dorsal view of posterior tergites **B** anal plate. Scale bars: 100 μm.

**Figure 27.**
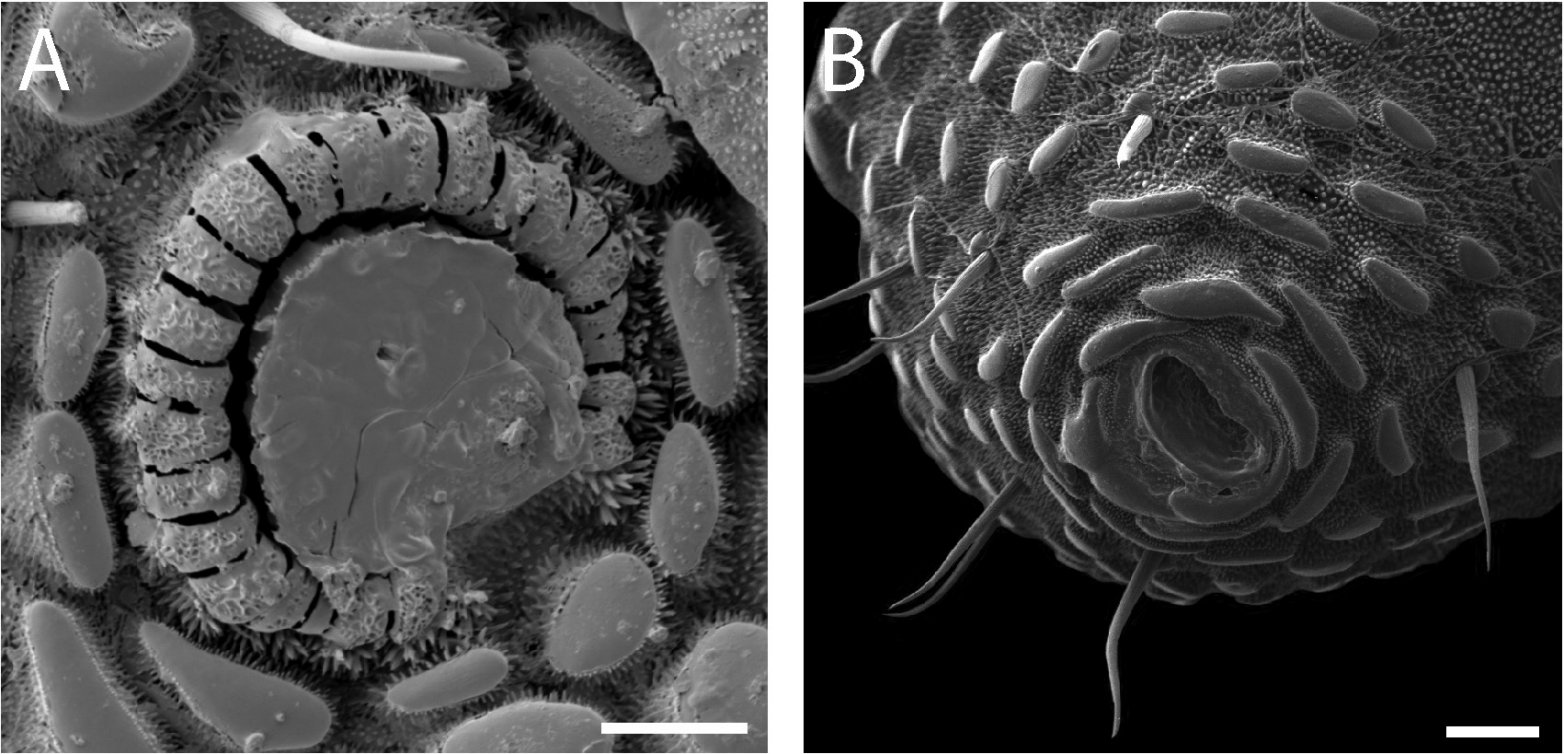
*Austropurcellia monteithi* sp. n., males. **A** spiracle, QM berlesate 836 **B** ozophore, QM 102443, paratype. Scale bars: 20 μm.

**Figure 28.**
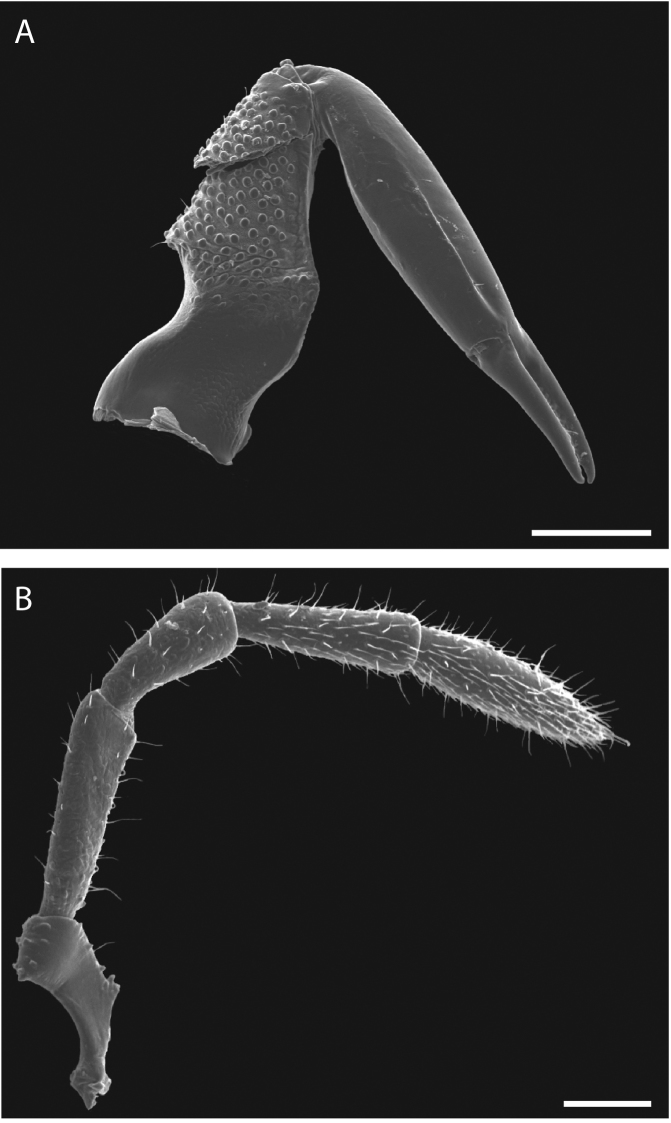
*Austropurcellia monteithi* sp. n., paratype male, QM 102443. **A** chelicera **B** palp. Scale bar: 200 μm (**A**); 200 μm (**B**).

**Figure 29.**
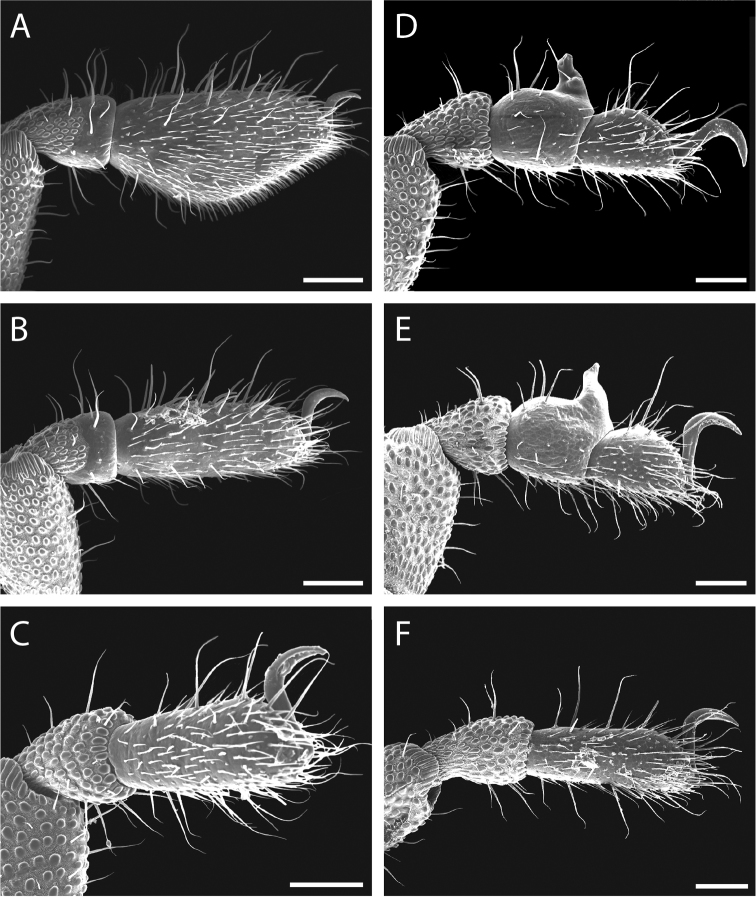
*Austropurcellia monteithi* sp. n., paratype male and female, QM 102443. **A** male tarsus and metatarsus I **B** male tarsus and metatarsus II **C** male tarsus and metatarsus III **D** male tarsus and metatarsus IV, lateral view **E** male tarsus and metatarsus IV, medial view **F** female tarsus and metatarsus IV. Scale bars: 100 μm.

Figs 24 , 25 , 26 , 27 , 28 , 29

##### Material examined.

*Holotype*. Male (QM 102442 [ex MCZ IZ 68951]), Kahlpahlim Rock (Lambs Head) Trail trailhead, Dinden National Park, 17.037°S, 145.613°E, coll. S. L. Boyer, M. J. Coblens, K. R. Jay and P. P. Sharma 30.v.2014.

*Paratypes*. 1 male, 1 female, same collecting data as holotype, QM 102443 (ex MCZ IZ 69023). 1 male, 1 female, same collecting data as holotype, MCZ IZ 69024, Macalester SEM stubs M28.9, M28.10, M30.3.

##### Additional material.

1 male, Mt. Edith Summit, 17.093°S, 145.622°E, coll. G. B. Monteith 8.iv.2014, MCZ IZ 69025.

1 male, Davies Creek Road, 17.050°S, 145.600°E, coll. G. B. Monteith and G. Thompson 17.xii.1989, QM berlesate 836, S 25699, Macalester SEM stubs M21.5, M21.6.

1 male, Chujeba Peak Summit, 16.936°S, 145.657°E, coll. G. B. Monteith and G. Thompson 14-16.xii.1989, QM S 41074, Macalester SEM stubs M21.1, M21.2.

4 males, 1 female, 1 juvenile, Mount Williams Summit, 16.917°S, 145.667°E, coll. G. B. Monteith 28.xi.1997, QM berlesate 962, S 35866, Macalester SEM stubs M19.9, M19.10.

1 male, 3 females, 1 juvenile, Mount Williams, 16.917°S, 145.667°E, coll. G. B. Monteith 28.xi.1997, QM berlesate 961, S 35868, Macalester SEM stubs M20.5, M20.6.

1 male, 1 female, 2 juveniles, Mount Williams, 16.917°S, 145.667°E, coll. G. B. Monteith and H. Janetzki 3.xii.1993, QM berlesate 867, S 49641, Macalester SEM stubs M20.7, M20.8.

##### Diagnosis.

Distinguished from congeners by an unusually wide scopula emerging from anterior margin or anterior quarter of male anal plate and covering entire width of anal plate. Closely resembles *Austropurcellia megatanka* sp. n., due to full scopula covering most of anal plate, but distinguished from *Austropurcellia megatanka* by differences in scopula shape and ubiquity of ornamentation on opisthosomal sternites.

##### Description.

Pettalid with tergite VIII bilobed (Fig. 25). Length of male holotype (Fig. 24) 2.0 mm, width at widest point in posterior third of prosoma 1.2 mm, width at ozophores 0.8 mm. Most of body surface covered in microstructure of tubercles and granules (Fig. 25). Transverse sulci present and granulated (Figs 25A, 26A). Medial sulcus present, oriented parallel to posterior-anterior axis, containing elongated granules oriented parallel to medial sulcus (Fig. 25A).

Ozophores tall and conical, of type III *sensu* Juberthie (1970) (Figs 25A, 27B). Coxae of legs I and II mobile, coxae of remaining legs fixed. Male coxae II–IV meeting in the midline (Fig. 25B). Male gonostome small, subtriangular, wider than long (Fig. 25B). Spiracles circular and C-shaped with slightly recurved edges (Fig. 27A), as found in “open circle” type of Giribet and Boyer (2002). Anal region of “pettalid type” (Giribet and Boyer 2002). Anal plate convex and sparsely granulated near anterior margin, with granulation density increasing laterally (Fig. 26B). Very wide scopula emerging from anterior quarter of anal plate or from anterior margin and continuing past posterior margin of anal plate (Fig. 26B). Two anal pores visible, one at suture between anal plate and tergite IX and one between lobes of tergite VIII (Fig. 26B).

Chelicerae (Fig. 28A) short and relatively robust. Proximal article of chelicerae with dorsal crest, without ventral process. Median article with apodeme. Chela with two types of dentition typical in pettalids (Fig. 28A). Measurements from male paratype of cheliceral articles from proximal to distal (in mm): 0.61, 0.83. Palp (Fig. 28B) with prominent ventral process on trochanter. Measurements from male paratype of palp articles from proximal to distal (in mm): 0.23, 0.28, 0.20, 0.23, 0.27.

Legs with all claws smooth, without ventral dentition or lateral pegs (Fig. 29). All tarsi smooth (Fig. 29). Distinct solea present on ventral surface of tarsus I (Fig. 29A). Metatarsi I and II heavily ornamented on proximal half, with distal half smooth (Fig. 29A, B). Remaining metatarsi with full ornamentation (Fig. 29C–F). Male tarsus IV fully divided into two tarsomeres (Fig. 29D, E). Adenostyle with relatively robust claw, wide base, and small pore at apex on lateral (external) side (Fig. 29D). Long seta rising from medial (internal) face of adenostyle from below pore to above apex (Fig. 29D, E); very short seta rising from adenostyle base below pore on lateral (external) face (Fig. 29D) (example with adenostyle features labeled, Fig. 5).

Measurements from male paratype of leg articles from proximal to distal (in mm): leg I 0.15, 0.50, 0.27, 0.38, 0.19, 0.43; leg II 0.17, 0.39, 0.19, 0.31, 0.12, 0.34; leg III [trochanter damaged], [femur damaged] 0.20, 0.25, 0.11, 0.27; leg IV [trochanter damaged], 0.41, 0.23, 0.33, 0.13, 0.37. Width measurements from male paratype of leg articles from proximal to distal (in mm): leg I [trochanter damaged], 0.18, 0.18, 0.17, 0.15, 0.22; leg II [trochanter damaged], 0.15, 0.16, 0.17, 0.13, 0.14; leg III [trochanter damaged], 0.18, 0.17, 0.18, 0.12, 0.13; leg IV [trochanter damaged], 0.20, 0.17, 0.19, 0.17, 0.16.

##### Etymology.

The specific epithet is a tribute to the legendary Queensland field biologist Geoff Monteith for his invaluable knowledge of Wet Tropics entomology, which guided much of our fieldwork. The authors also wish to recognize his outsize generosity and hospitality to visiting researchers. In addition, he collected many of the specimens used in this study, including the holotype for *Austropurcellia monteithi* sp. n.

#### Austropurcellia nuda

Taxon classificationAnimaliaOpilionesPettalidae

Popkin-Hall, Jay & Boyer sp. n.

http://zoobank.org/1E45BE1D-2DA4-47AD-BAC6-063C417E05DC

**Figure 30.**
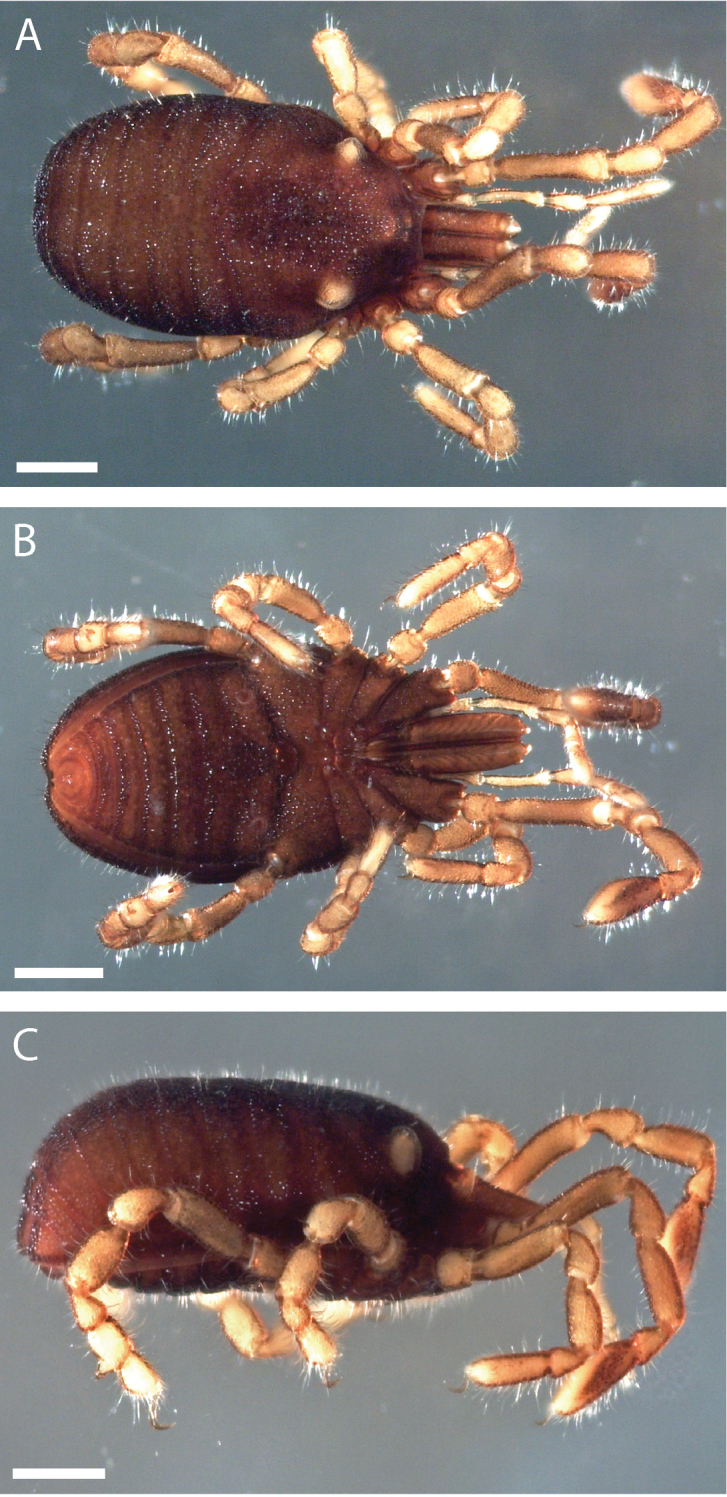
*Austropurcellia nuda* sp. n., holotype male, QM 102444. **A** dorsal view **B** ventral view **C** lateral view. Scale bars: 0.5 mm.

**Figure 31.**
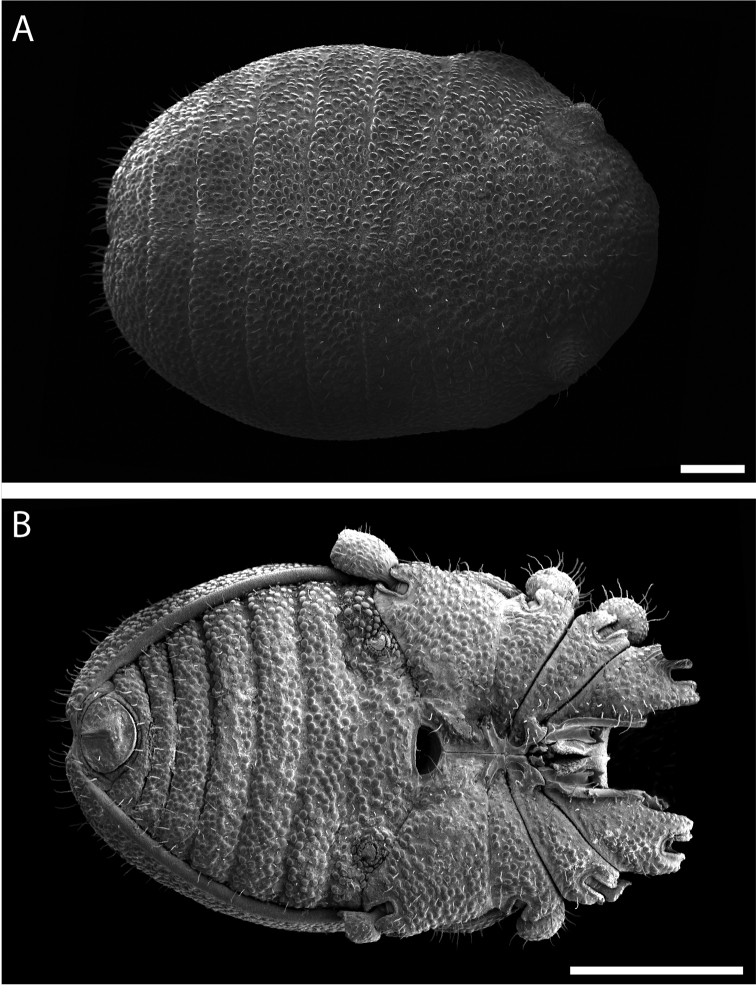
*Austropurcellia nuda* sp. n., males. **A** dorsal view, QM berlesate 413 **B** ventral view, QM berlesate 38118, paratype. Scale bar: 200 μm (**A**); 500 μm (**B**).

**Figure 32.**
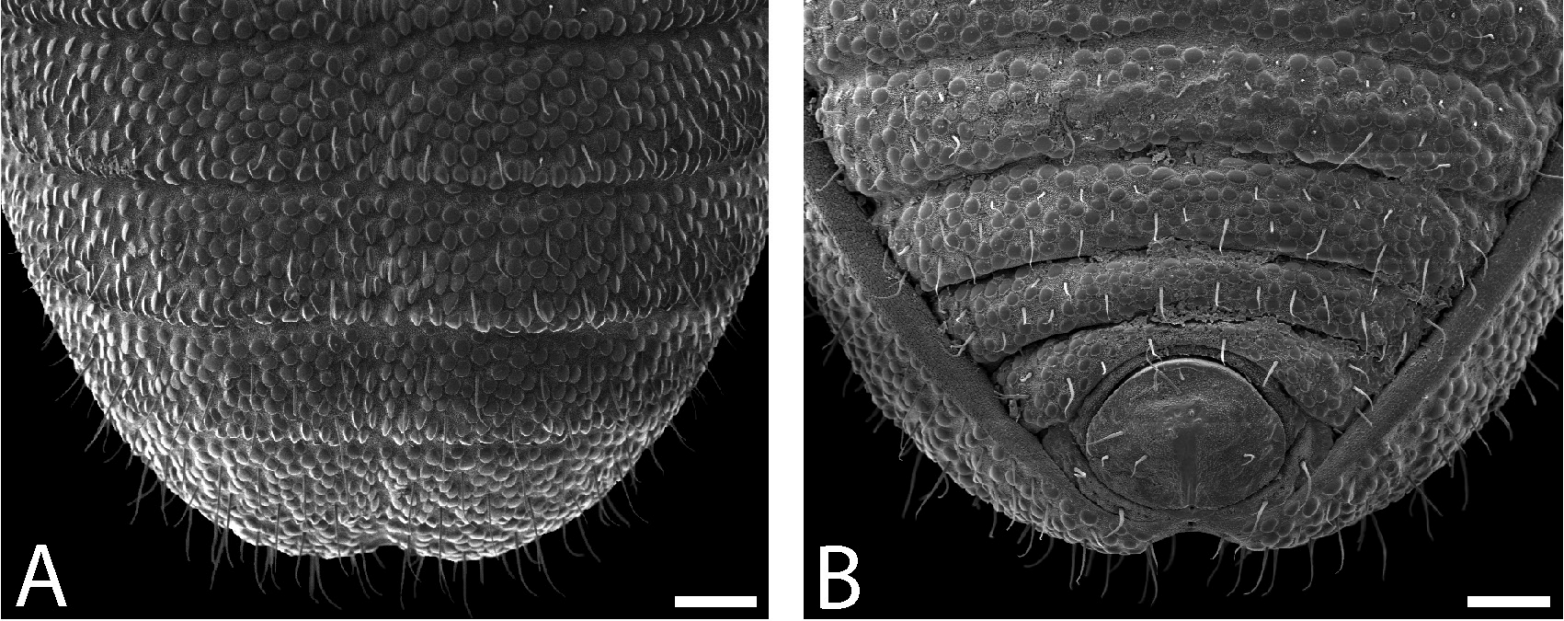
*Austropurcellia nuda* sp. n., males. **A** dorsal view of posterior tergites, QM berlesate 38118, paratype **B** anal plate, QM berlesate 413. Scale bars: 100 μm.

**Figure 33.**
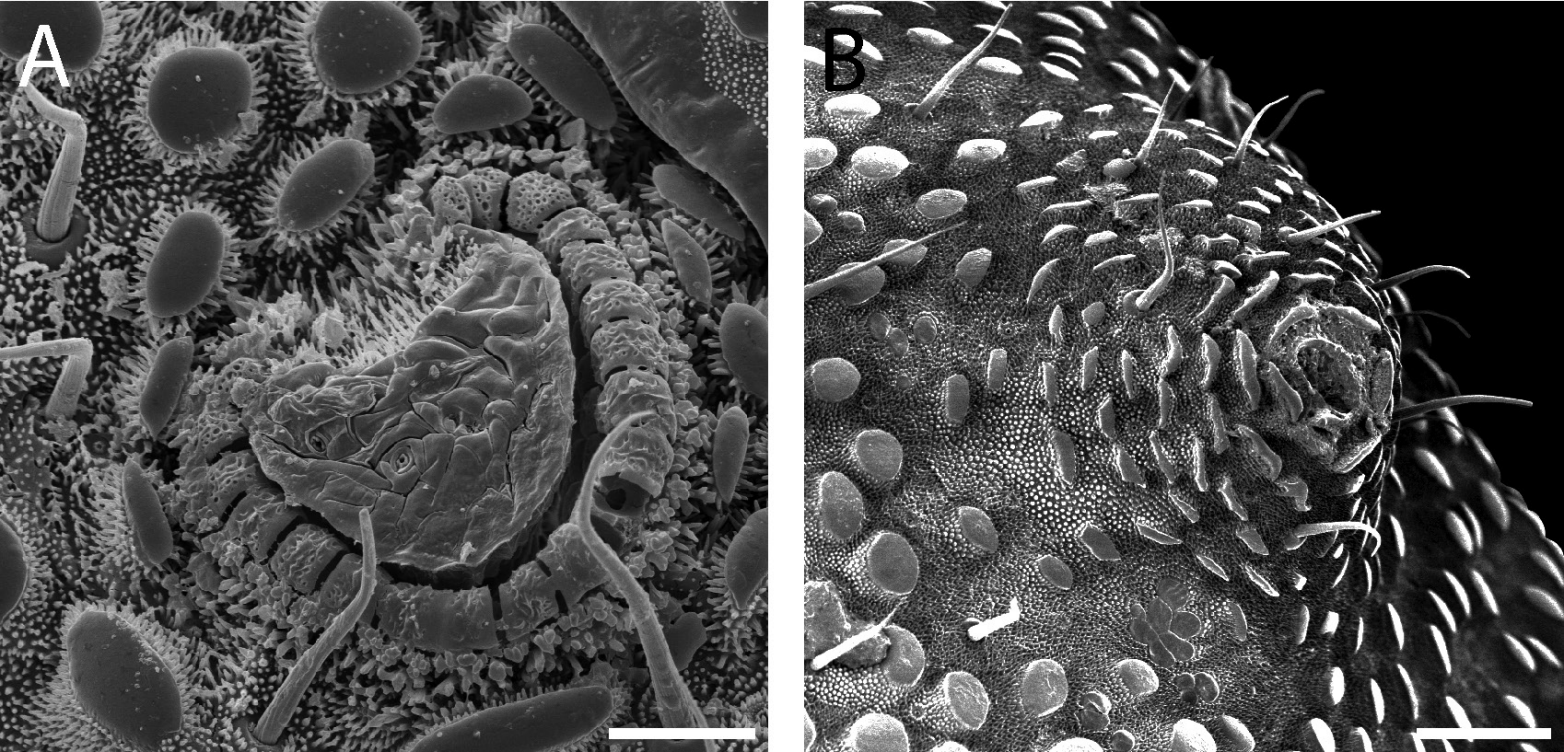
*Austropurcellia nuda* sp. n., males. **A** spiracle, QM berlesate 413 **B** ozophore, QM berlesate 38118, paratype. Scale bar: 20 μm (**A**); 50 μm (**B**).

**Figure 34.**
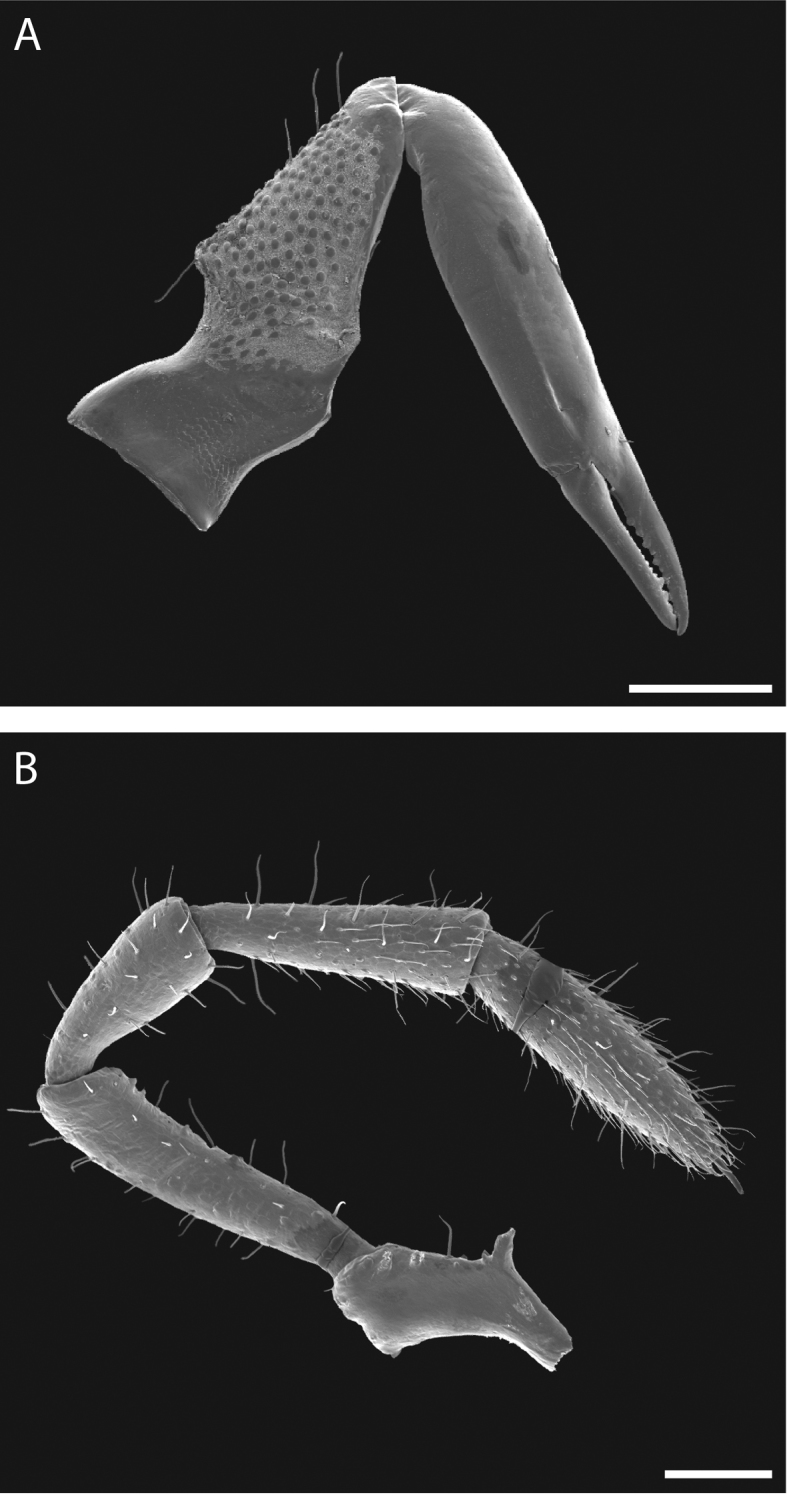
*Austropurcellia nuda* sp. n., males, QM berlesate 413. **A** chelicera **B** palp. Scale bar: 200 μm (**A**); 100 μm (**B**).

**Figure 35.**
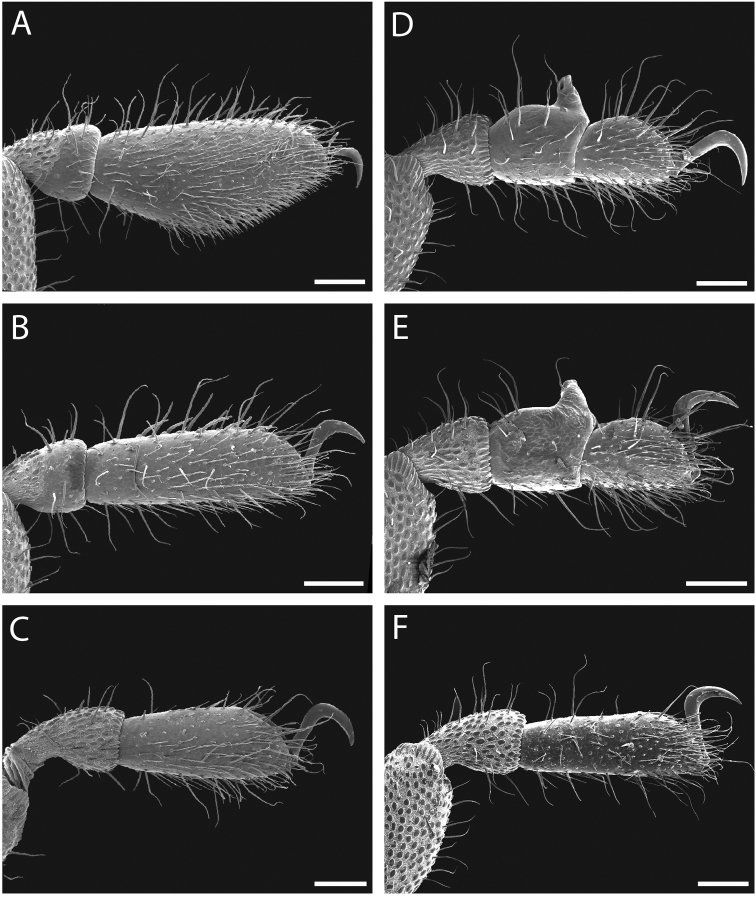
*Austropurcellia nuda* sp. n., males and female. **A** male tarsus and metatarsus I, QM berlesate 413 **B** male tarsus and metatarsus II, QM berlesate 413 **C** male tarsus and metatarsus III, QM berlesate 413 **D** male tarsus and metatarsus IV, lateral view, QM berlesate 38118, paratype **E** male tarsus and metatarsus IV, medial view, QM berlesate 38118, paratype **F** female tarsus and metatarsus IV, QM berlesate 413. Scale bars: 100 μm.

Figs 30 , 31 , 32 , 33 , 34 , 35

##### Material examined.

*Holotype*. Male (QM 102444 [ex QM 38118]), Black Mountain Summit, 16.644°S, 145.49°E, coll. K. Aland and G. B. Monteith 30.iv.2015, QM 38118.

*Paratypes*. 10 males, 19 females, 24 juveniles, same collecting data as holotype, QM 38118, Macalester SEM stubs M30.4, M30.8, M30.9.

##### Additional material.

2 males, 2 females, Black Mountain 17 km ESE Julatten, 16.650°S, 145.483°E, coll. G. B. Monteith, D. Yeates, and D. Cook 29.iv.1982, S 2302, QM berlesate 413, Macalester SEM stubs M20.1, M20.2.

##### Diagnosis.

Distinguished from congeners by lack of scopula on the male anal plate, a trait shared only with *Austropurcellia absens*. Anal plate is flat and entirely ungranulated; *Austropurcellia absens* anal plate is convex, bilobed, and mostly granulated.

##### Description.

Pettalid with tergite VIII bilobed (Fig. 31). Length of male holotype (Fig. 30) 2.1 mm, width at widest point in posterior third of prosoma 1.2 mm, width at ozophores 0.8 mm. Most of body surface covered in microstructure of tubercles and granules (Fig. 31). Transverse sulci present and distinct by lack of granulation (Figs 31A, 32B). Dorsal longitudinal sulcus containing elongated granules oriented flanking dorsal longitudinal sulcus (Figs 31A, 32B). Sternites ubiquitously granulated (Fig. 31B).

Ozophores relatively conical, of type III *sensu* Juberthie (1970) (Figs 31A, 33B). Coxae of legs I and II mobile, coxae of remaining legs fixed. Male coxae II–IV meeting in the midline (Fig. 31B). Male gonostome small, subtriangular, and wider than long (Fig. 31B). Spiracles circular and C-shaped with slightly recurved edges (Fig. 33A), as found in “open circle” type of Giribet and Boyer (2002). Anal region of “pettalid type” (Giribet and Boyer 2002). Male tergite VIII bilobed, with lobes ornamented dorsally and ventrally (Fig. 32). Anal plate relatively flat and ungranulated over entire surface with narrow, smooth area running vertically through center from just above the center down to the posterior margin (Fig. 32B). Scopula absent (Fig. 32B). Two anal pores visible (Fig. 32B).

Chelicerae (Fig. 34A) short and relatively robust. Proximal article of chelicerae with dorsal crest, without ventral process. Median article with prominent apodeme. Chela with two types of dentition typical in pettalids (Fig. 34A). Measurements of cheliceral articles of male paratype from proximal to distal (in mm): 0.61, 0.84, 0.28. Palp (Fig. 34B) with prominent ventral process on trochanter. Measurements of palpal articles of male paratype from proximal to distal (in mm): 0.20, 0.29, 0.18, 0.24, 0.27.

Legs with all claws smooth, without ventral dentition or lateral pegs (Fig. 35). All tarsi smooth (Fig. 35). Distinct solea present on ventral surface of tarsus I (Fig. 35A). Metatarsi I and II heavily ornamented on proximal half, with distal half smooth (Fig. 35A, B). Remaining metatarsi with full ornamentation (Fig. 35C-F). Male tarsus IV fully divided into two tarsomeres (Fig. 35D, E). Adenostyle with robust claw, wide base, and small pore at apex on lateral (external) side (Fig. 35D). Long seta rising from medial (internal) face of adenostyle from below pore to above apex (Fig. 35D, E); very short seta rising from adenostyle base below pore on lateral (external) face (Fig. 35D) (example with adenostyle features labeled, Fig. 5).

Measurements from male paratype of leg articles from proximal to distal (in mm): leg I 0.18, 0.52, 0.20, 0.39, 0.14, 0.41; leg II 0.15, 0.41, 0.19, 0.33, 0.14, 0.37; leg III [trochanter damaged], 0.37, 0.20, 0.29, 0.23, 0.32; leg IV 0.19, 0.44, 0.19, 0.35, 0.16, 0.39. Width measurements from male paratype of leg articles from proximal to distal (in mm): leg I 0.20, 0.16, 0.17, 0.16, 0.14, 0.20; leg II 0.18, 0.15, 0.15, 0.15, 0.12, 0.13; leg III [trochanter damaged], 0.17, 0.16, 0.19, 0.13, 0.14; leg IV 0.18, 0.19, 0.18, 0.18, 0.15, 0.17.

##### Etymology.

The specific epithet is derived from the first declension form of *nūdus*, from Latin, meaning “bare” or “naked”, a reference to diagnostic absence of the scopula or ornamentation of the anal plate in this species.

#### Austropurcellia riedeli

Taxon classificationAnimaliaOpilionesPettalidae

Jay, Oberski & Boyer sp. n.

http://zoobank.org/672A8F03-19C4-47CE-BD10-85156747B57F

**Figure 36.**
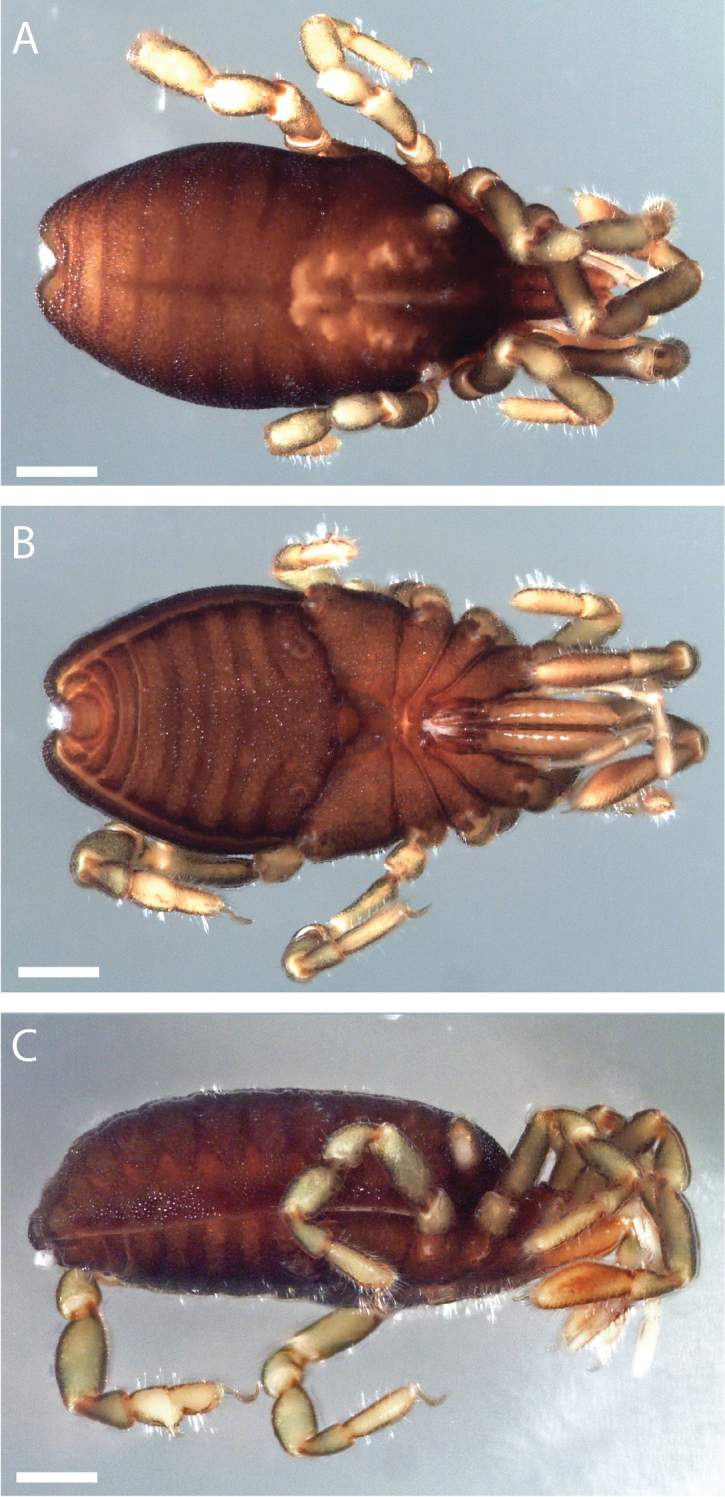
*Austropurcellia riedeli* sp. n., holotype male, QM 102448. **A** dorsal view **B** ventral view **C** lateral view. Scale bars: 0.5 mm.

**Figure 37.**
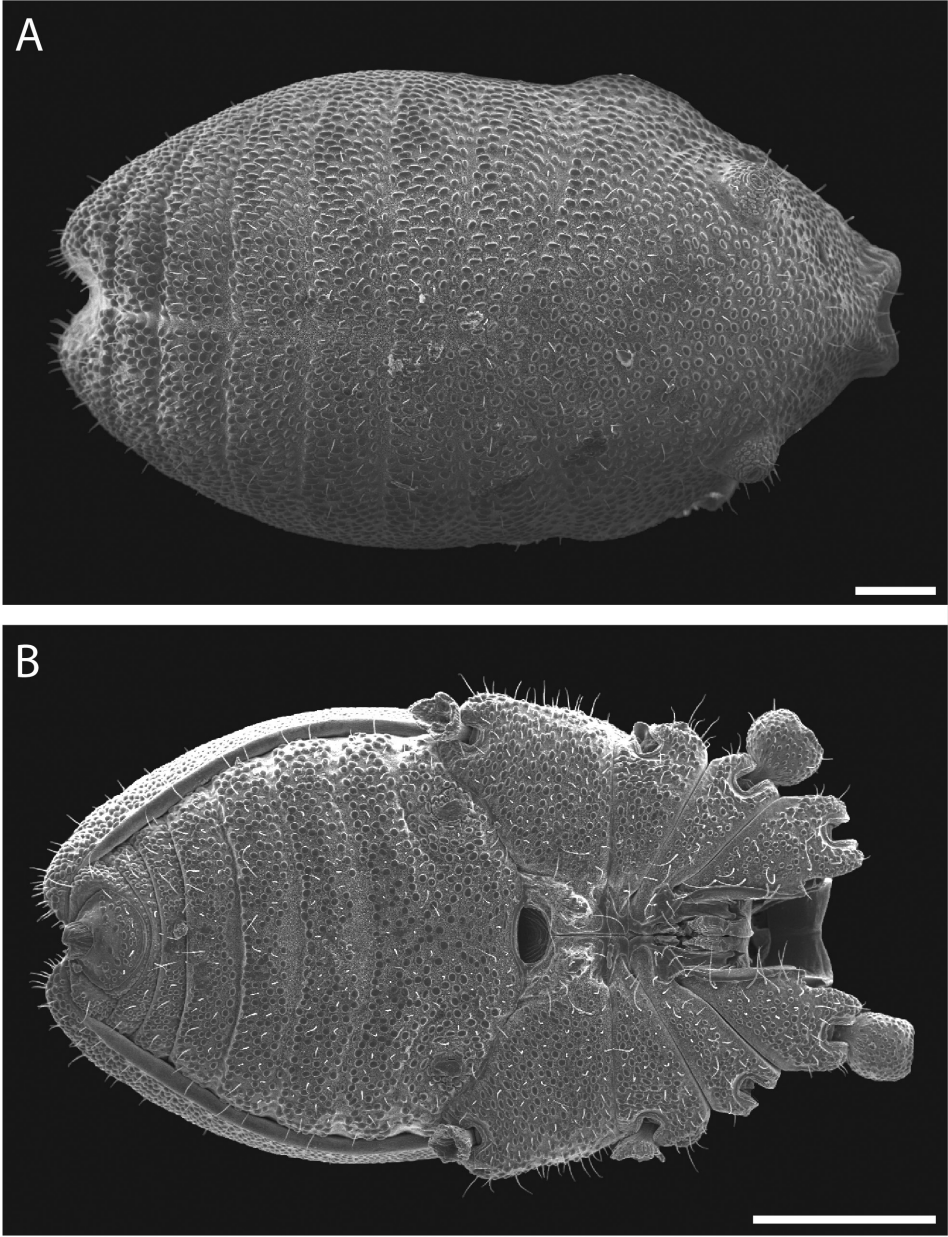
*Austropurcellia riedeli* sp. n., male, MCZ IZ 69026. **A** dorsal view **B** ventral view. Scale bar: 200 μm (**A**); 500 μm (**B**).

**Figure 38.**
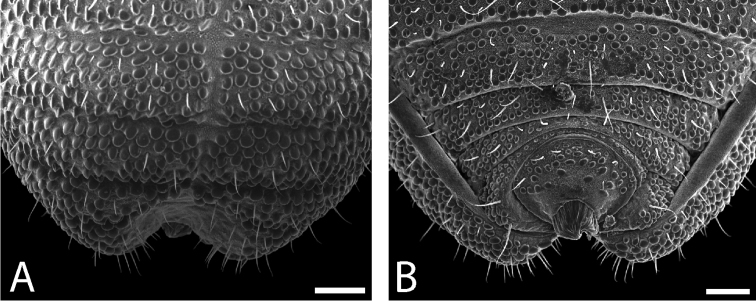
*Austropurcellia riedeli* sp. n., male, MCZ IZ 69026. **A** dorsal view of posterior tergites **B** anal plate. Scale bars: 100 μm.

**Figure 39.**
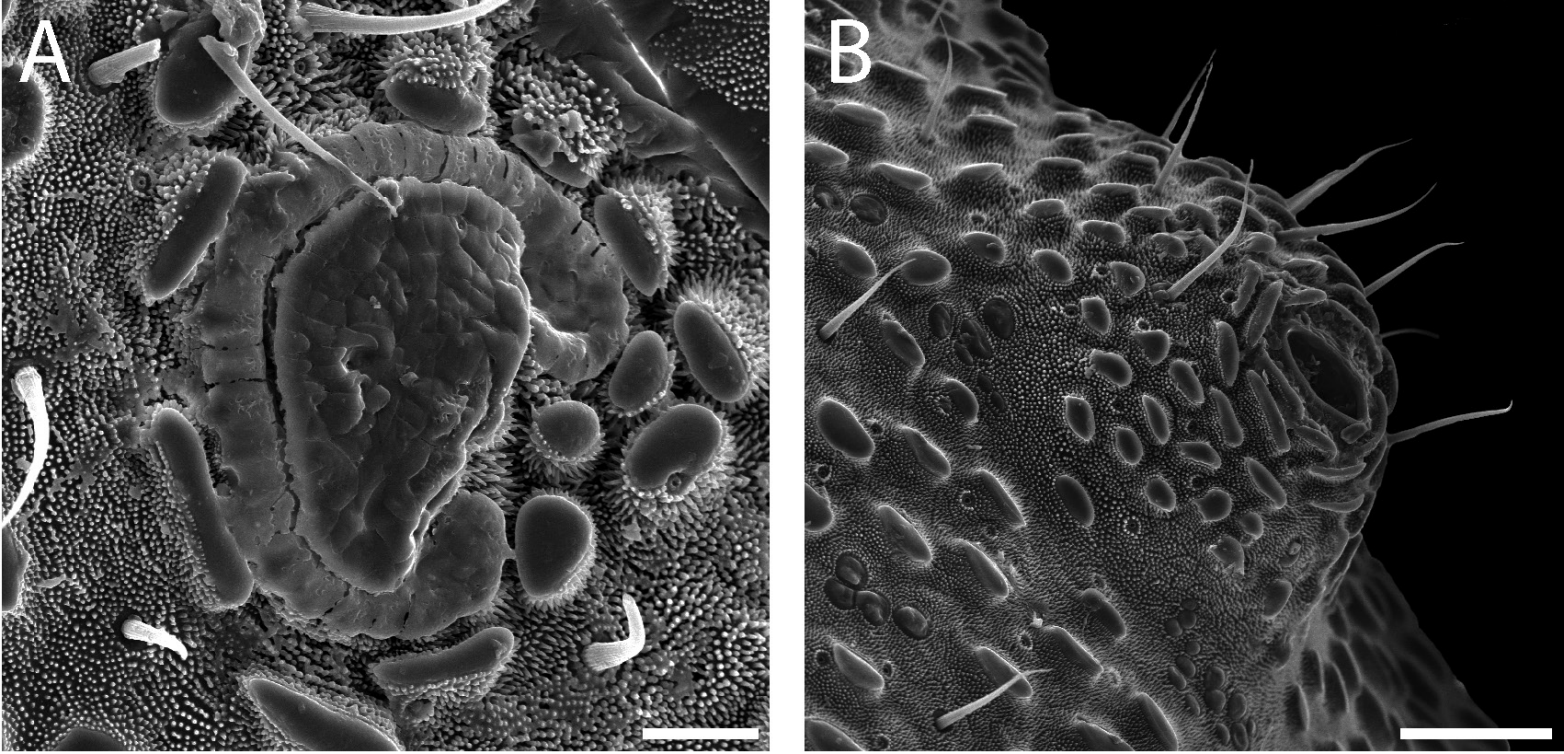
*Austropurcellia riedeli* sp. n., male, MCZ IZ 69026. **A** spiracle **B** ozophore. Scale bar: 20 μm (**A**); 50 μm (**B**).

**Figure 40.**
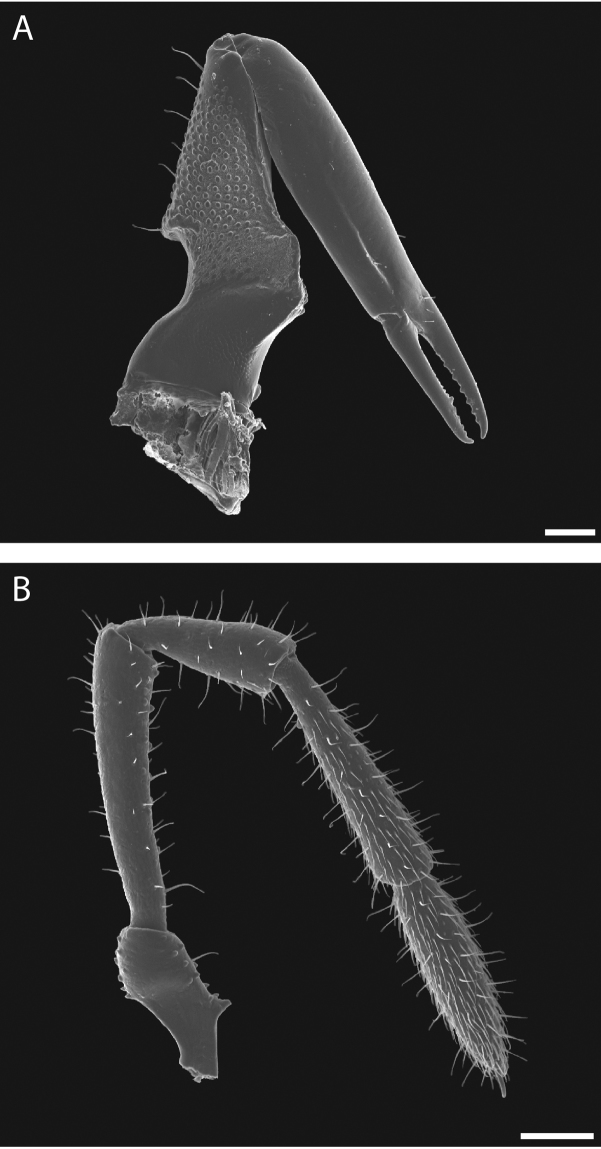
*Austropurcellia riedeli* sp. n., male, MCZ IZ 69026. **A** chelicera **B** palp. Scale bars: 100 μm.

**Figure 41.**
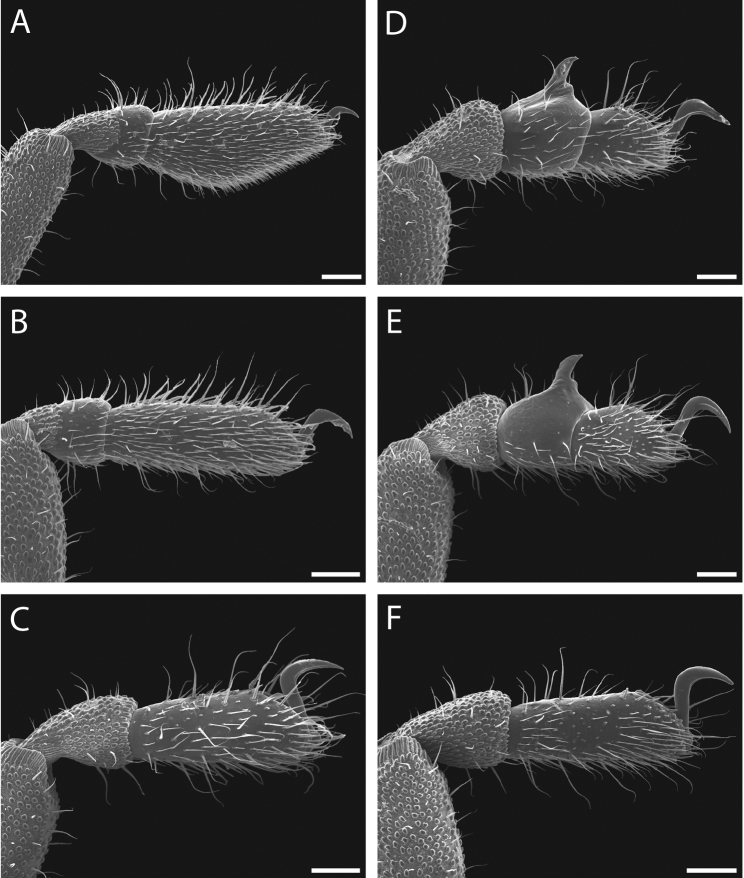
*Austropurcellia riedeli* sp. n., male and female, MCZ IZ 69026. **A** male tarsus and metatarsus I **B** male tarsus and metatarsus II **C** male tarsus and metatarsus III **D** male tarsus and metatarsus IV **E** male tarsus and metatarsus IV **F** female tarsus and metatarsus IV. Scale bars: 100 μm.

Figs 36 , 37 , 38 , 39 , 40 , 41

##### Material examined.

*Holotype*. Male (QM 102448), Rossville, Bloomfield (sample 2B, AR2), 15.792°S, 145.302°E, coll. Alex Riedel 1.v.2014.

*Paratype*. 1 female, Rossville, Bloomfield (sample 2B, AR2), 15.792°S, 145.302°E, coll. Alex Riedel 1.v.2014, QM 102449.

##### Additional material.

1 male, 2 females, Rossville, Bloomfield (sample 2, AR1), 15.792°S, 145.302°E, coll. Alex Riedel 29.iv.2014, MCZ IZ 69026, Macalester SEM stubs M30.13, M30.14, M30.15.

##### Diagnosis.

Distinguished from congeners by flat anal plate granulated anteriorly, with very short and round scopula emerging from posterior third of plate. Lobes of tergites VIII and IX rounded and prominent in dorsal view; absence of granulation in junction of the anal plate, the lobes of tergite VIII, and the dorsal scutum. Closely resembles *Austropurcellia finniganensis*, but distinguished by its larger body size (0.4 mm longer, 0.2 mm wider).

##### Description.

Pettalid with tergite VIII bilobed (Fig. 37). Posterior margin of dorsal scutum curves ventrally (Fig. 36C). Length of male holotype (Fig. 36) 2.5 mm, width at widest point in posterior third of prosoma 1.4 mm, width at ozophores 1.0 mm. Most of body surface covered in microstructure of tubercles and granules (Fig. 37). Transverse sulci present and distinct by lack of granulation (Figs 36A, 37A). Dorsal longitudinal sulcus lacking granulation, with adjacent flanking granules oriented parallel to dorsal longitudinal sulcus (Figs 36A, 37A). Anterior edge of sternites IV and V lacking granulation medially (Fig. 37B).

Ozophores relatively conical, of type III *sensu* Juberthie (1970) (Fig. 37A, 39B). Coxae of legs I and II mobile; coxae of remaining legs fixed. Male coxae II-IV meeting in the midline (Fig. 37B). Male gonostome small, subtriangular, wider than long (Fig. 37B). Spiracles circular and C-shaped with slightly recurved edges (Fig. 39A), as found in “open circle” type of Giribet and Boyer (2002). Anal region of “pettalid type” (Giribet and Boyer 2002). Anal plate flat, posteriorly convex, with anterior granulation (Fig. 38B). Short, round scopula extruding from circular area on posterior third of anal plate and extending just past posterior margin of anal plate (Fig. 38B). Orientation of scopula obscures anal pores, which are not visible (Fig. 38B).

Chelicerae (Fig. 40A) short and relatively robust. Proximal article of chelicerae with dorsal crest, without ventral process. Median article with prominent apodeme. Chela with two types of dentition typical in pettalids (Fig. 40A). Measurements of cheliceral articles of male paratype from proximal to distal (in mm): 0.81, 1.02, 0.33. Palp (Fig. 40B) with prominent ventral process on trochanter. Measurements from palpal articles of male paratype from proximal to distal (in mm): 0.22, 0.39, 0.21, 0.35, 0.29.

Legs with all claws smooth, without ventral dentition or lateral pegs (Fig. 41). All tarsi smooth (Fig. 41). Distinct solea present on ventral surface of tarsus I (Fig. 41A). Metatarsi I and II heavily ornamented on proximal half, with distal half smooth (Fig. 40A, B). Remaining metatarsi with full ornamentation (Fig. 41C–F). Male tarsus IV fully divided into two tarsomeres (Fig. 41D, E). Adenostyle with relatively robust, pointed claw curving distally, wider base, and small pore at apex on lateral (external) side (Fig. 41D). Long seta rising from medial (internal) face of adenostyle from below pore to above apex (Fig. 41D, E); very short seta rising from adenostyle base below pore on lateral (external) face (Fig. 41D) (example with adenostyle features labeled, Fig. 5).

Measurements from male paratype of leg articles from proximal to distal (in mm): leg I 0.28, 0.64, 0.25, 0.48, 0.23, 0.46; leg II 0.22, 0.49, 0.25, 0.39, 0.16, 0.42; leg III 0.17, 0.42, 0.25, 0.35, 0.20, 0.39; leg IV 0.22, 0.61, 0.29, 0.44, 0.25, 0.41. Width measurements from male paratype of leg articles from proximal to distal (in mm): leg I 0.23, 0.21, 0.21, 0.20, 0.17, 0.23; leg II 0.21, 0.19, 0.19, 0.20, 0.15, 0.15; leg III 0.22, 0.21, 0.20, 0.20, 0.15, 0.16; leg IV 0.25, 0.23, 0.24, 0.25, 0.21, 0.20.

##### Etymology.

This species is named after Alex Riedel, the German entomologist who provided us with several collections of animals from key localities, including Mount Finnigan.

## Discussion

To develop *Austropurcellia* further as a system for studying WT biodiversity and biogeography, it is critical to describe and map the diversity of these cryptic, dispersal-limited arachnids. Significant progress has been made toward this aim; with the six new *Austropurcellia* species described here in addition to other recent work (Boyer and Reuter 2012, Popkin-Hall and Boyer 2014, Boyer et al. 2015), the majority of mite harvestman diversity throughout Queensland is presently thought to be documented. These new species bring the total number of described species within *Austropurcellia* to 25, 21 of which are found within the WT biodiversity hotspot (Fig. 1).

With a greater knowledge of the diversity of *Austropurcellia* comes greater challenge in diagnosing the genus, as previously discussed by Popkin-Hall and Boyer (2014). In their 2007 phylogenetic analysis of the family Pettalidae, Boyer and Giribet re-diagnosed *Austropurcellia* to include the presence of a scopula in the anal plate. However, we now know of two species of *Austropurcellia* that lack a scopula in the anal plate, *Austropurcellia absens* (Fig. 44E) and *Austropurcellia nuda* sp. n. That said, the most recent phylogenetic study of the genus indicates that this loss is secondary (i.e. not the ancestral state). The re-diagnosis identified the robustness of the adenostyle, with height no more than twice base length, as an important feature uniting *Austropurcellia*. Although this is present in many species (e.g. *Austropurcellia daviesae*, Fig. 45C), there are also species within the genus that have as thin, bladelike adenostyles (e.g. *Austropurcellia acuta*, Fig. 45F), and in this case the diagnostic character is likely ancestral with respect to *Austropurcellia*. Other diagnostic characters of Boyer and Giribet (2007) are still valid, including prominent ventral process on trochanter of the palp, lack of robust ventral process on the chelicera, solea in tarsus I, and male tarsus IV bisegmented dorsally to fully bisegmented (Fig. 45). However, all of these characters are shared with another lineage of pettalids, the New Zealand genus *Rakaia*. Several phylogenetic analyses of the family Pettalidae have indicated that these two genera are reciprocally monophyletic (Boyer and Giribet 2007, Boyer and Giribet 2009, Giribet et al. 2012, Boyer et al. 2015). However, those same analyses have remained equivocal about the relationship of *Austropurcellia* and *Rakaia* to each other. While there is some suggestion that they may be sister taxa (e.g. Boyer et al. 2015), support for that hypothesis is low and studies with extensive taxon sampling across the genus have suggested other possible relationships, also with low support (e.g. Giribet et al. 2012). Regardless of the relationship of *Austropurcellia* to other pettalids, it is clearly reciprocally monophyletic with all other genera. Therefore, we are left with a genus that is valid based on molecular characters and the phylogenetic criterion of monophyly, but currently lacks a robust diagnosis grounded in anatomy. Fortunately, *Austropurcellia* does not co-occur geographically with any other pettalid genus.

**Figure 42.**
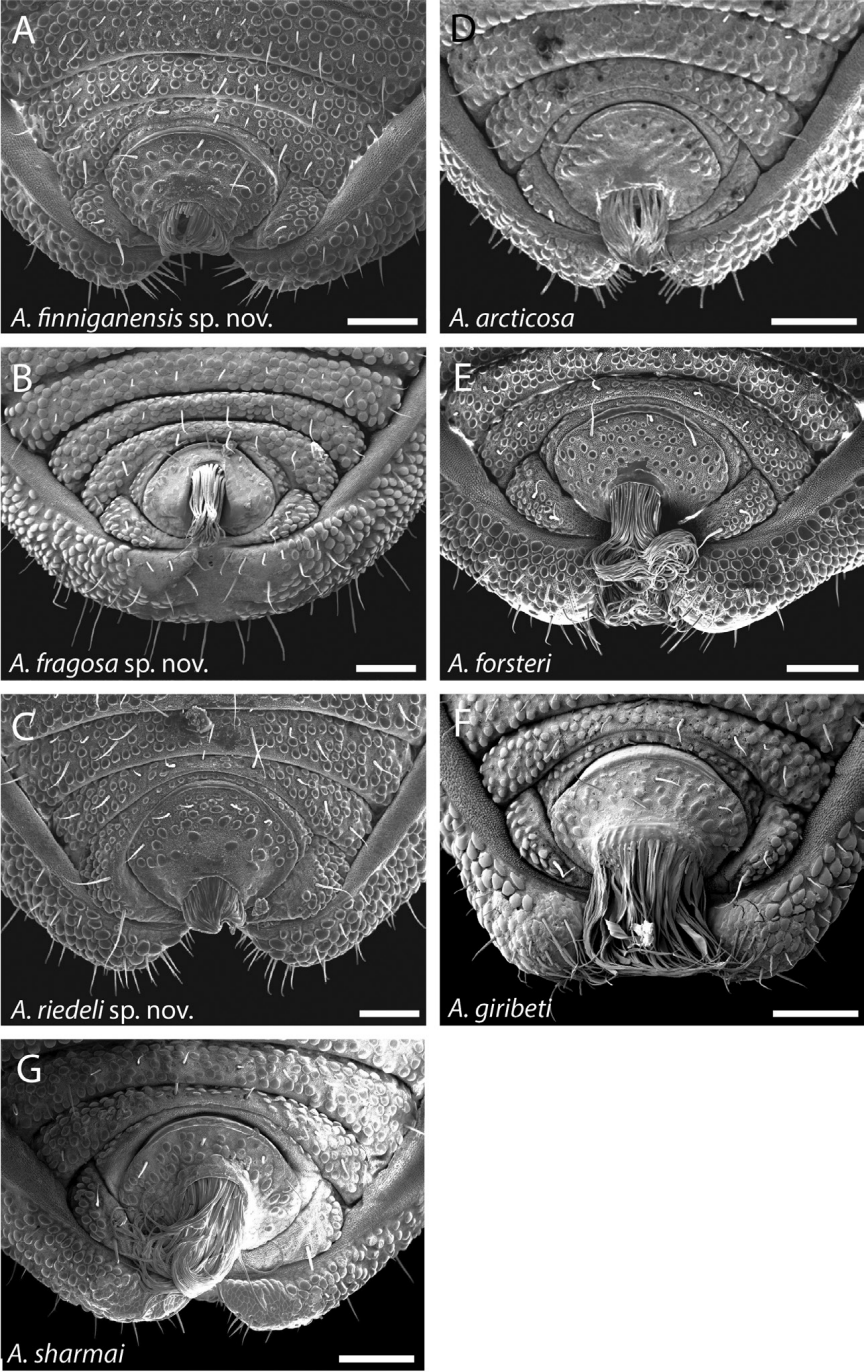
Ventral posterior morphology and anal plate of all seven species from the northernmost WT. Three new species are shown on left, with three previously described species on right. **A** *Austropurcellia finniganensis* from Mt. Finnigan, QM 102447 (paratype) **B** *Austropurcellia fragosa* from Roaring Meg Creek, QM berlesate 448 **C** *Austropurcellia riedeli* from Rossville, Boomfield, MCZ IZ 69026 **D** *Austropurcellia arcticosa*, from Dubuji Boardwalk, MCZ IZ 132327 **E** *Austropurcellia forsteri* from Cape Tribulation Top Camp, QM berlesate 486 **F** *Austropurcellia giribeti* from Alexandra Range, QM berlesate 252 **G** *Austropurcellia sharmai* from Emmagen Creek, MCZ IZ 132317. Scale bars: 100 μm.

**Figure 43.**
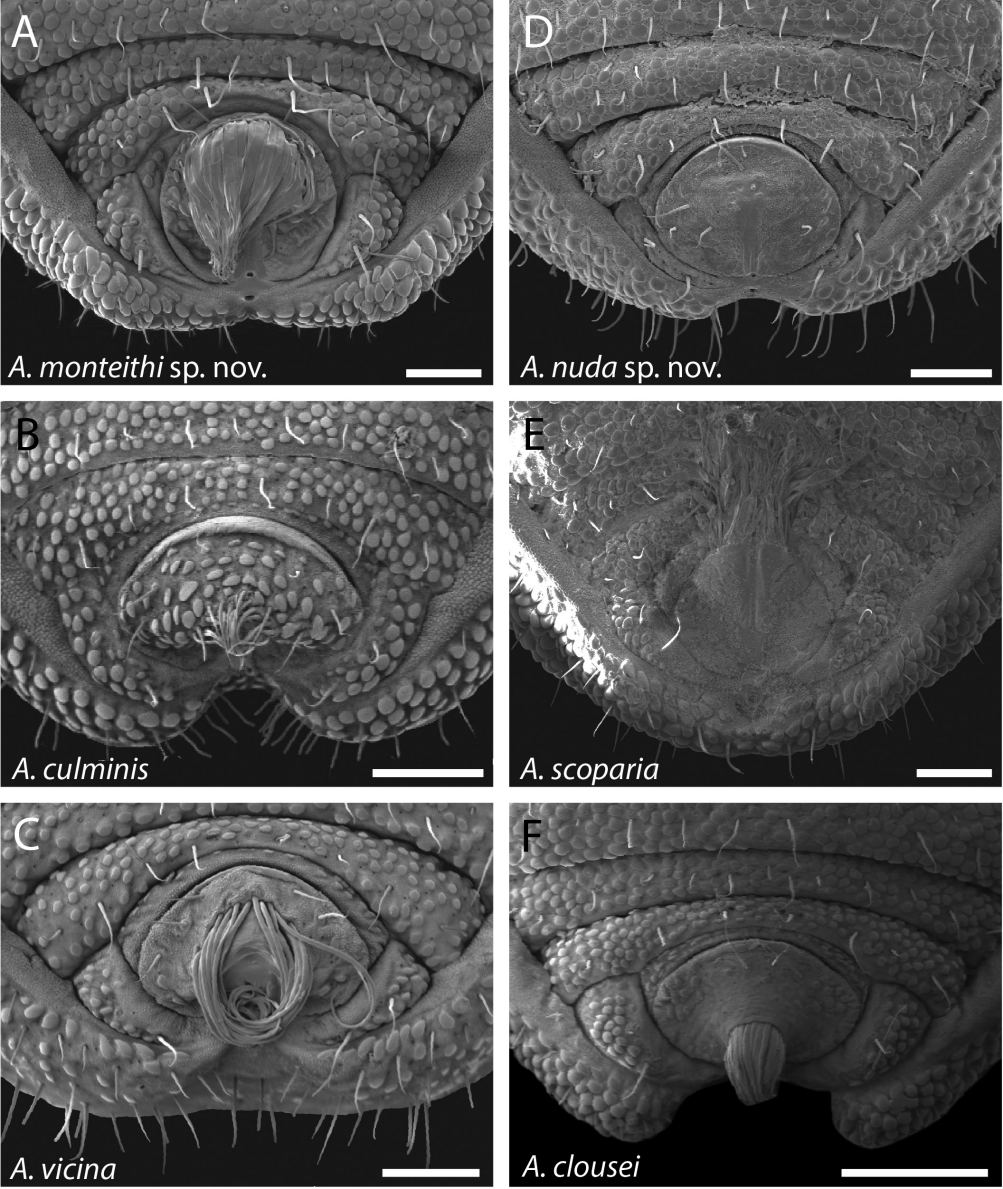
Ventral posterior morphology and anal plate of all five species from the north-central WT and one species from the southern WT (*Austropurcellia clousei*). Two new species are shown on top row, with four previously described species below. **A** *Austropurcellia monteithi*, from Kahlpahlim Rock (Lambs Head) Trail trailhead, QM 102443 (paratype) **B** *Austropurcellia culminis* from Bellenden Ker Summit, MHNG **C** *Austropurcellia vicina* from Crystal Cascades, ANIC berlesate 679 **D** *Austropurcellia nuda*, from Black Mountain, QM berlesate 38118 **E** *Austropurcellia scoparia* from Mt Spurgeon, QM S35834 **F** *Austropurcellia clousei* from Paluma Range National Park, MCZ IZ 132339 (paratype). Scale bars: 100 μm.

**Figure 44.**
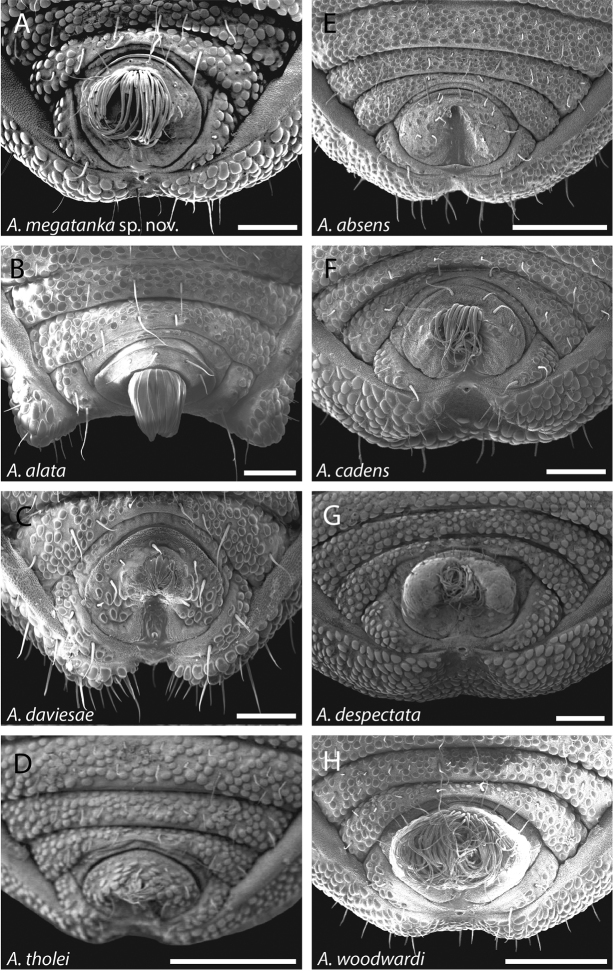
Ventral posterior morphology and anal plate of all eight species from the central WT uplands. One new species is shown alongside seven previously described species. **A** *Austropurcellia megatanka* from Baldy Mountain Road, QM 102441 (paratype) **B** *Austropurcellia alata* from Upper Boulder, QM berlesate 829 **C** *Austropurcellia daviesae* from Ella Bay, MCZ IZ 132343 **D** *Austropurcellia tholei* from Cathedral Fig. Tree, Danville State Forest, MCZ IZ 132330 (paratype) **E** *Austropurcellia absens* from Range Road, Kirrama Range, MCZ IZ 132316 (paratype) **F** *Austropurcellia cadens* from Mt. Bartle Frere, CAS HW0038 **G** *Austropurcellia despectata* from Millaa Millaa, ANIC berlesate 674 **H** *Austropurcellia woodwardi* from Boulder Creek, QM 1742. Scale bar: 100 μm (**A, B, C, F, G**); 200 μm (**H**); 250 μm (**D**).

**Figure 45.**
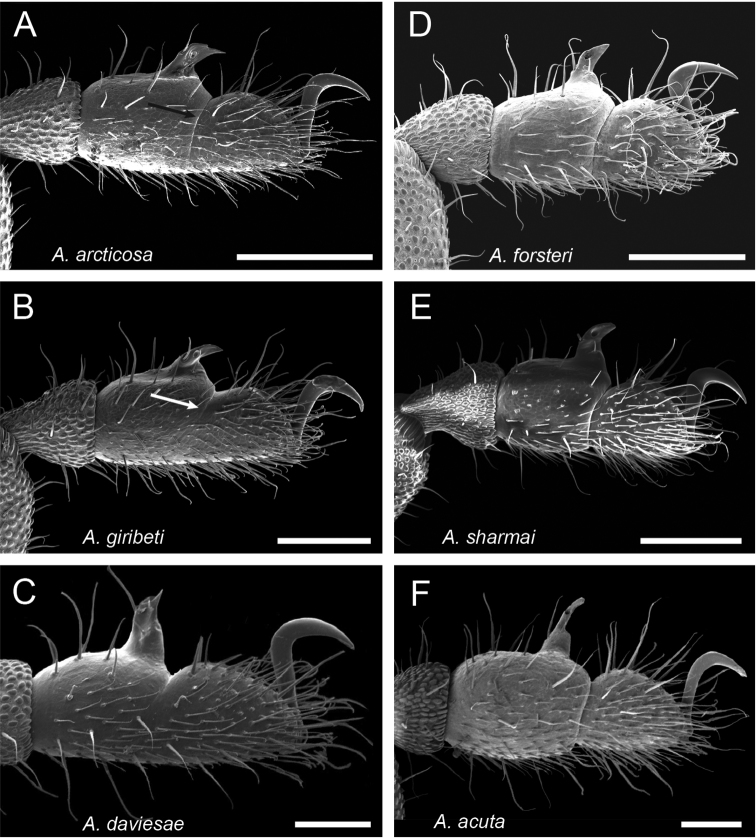
Fourth tarsus of male in lateral (external) view. **A** *Austropurcellia articosa* from Dubuji Boardwalk, MCZ IZ 132327 **B** *Austropurcellia giribeti* from Daintree Village, MCZ IZ 132344 **C** *Austropurcellia daviesae* from Ella Bay, MCZ DNA 106378 **D** *Austropurcellia forsteri* from Cape Tribulation Top Camp, QM berlese 486 **E** *Austropurcellia sharmai* from Emmagen Creek, MCZ IZ 132698 **F** *Austropurcellia acuta* from Bonyee Walk, MCZ IZ 132701. Scale bar: 100 μm (**A, B, C, F**); 250 μm (**D, E**).

Differences in morphological features within the genus can provide insight into evolutionary relationships, especially in the context of geographic distributions of character states. For example, Popkin-Hall and Boyer (2014) described the geographic variation of two morphological features: adenostyle shape and posterior lobe shape in tergite VIII, and found that all species from the WT tend to have a robust, blunt adenostyle morphology (e.g. Fig. 45A, D, E, C), while those south of the WT possess a thin, blade-like adenostyle shape (e.g. Fig. 5F). The six new species described herein are all distributed within the WT, and accord with the pattern of robust, blunt adenostyles being geographically concentrated in the northern end of *Austropurcellia*’s range. Previous work also demonstrates that male body shape varies with geography; species within the WT tend to have a rounded posterior, in contrast to the more triangular posterior and pointed lobes of tergite VIII found in species south of the WT. The six new species we describe support the pattern, as they all have rounded posterior lobes like those found in previously described WT species (Figs 7, 13, 19, 25, 31, 37, 42, 43, 44). However, a handful of species arguably defy this trend—*Austropurcellia giribeti* and *Austropurcellia articosa* (Fig. 42D, F), both found in the northern WT, share the more triangular posterior shape typically found in species south of the WT, while *Austropurcellia superbensis* from Southeast Queensland has a more rounded posterior shape.

Further defining characters that vary significantly between species include the size, position, and shape of both the anal plate and the scopula (Figs 42–44). The anal plate is flat in some species and convex to a variable degree in others. Scopula morphology varies in terms of its position on the anal plate and its size; in some species it emerges from the anterior portion of the anal plate while in others it emerges near the posterior margin of the anal plate. Species found in the northernmost WT tend to have a long, narrow scopula that emerges from the posterior end of the anal plate and is oriented parallel to the body (Fig. 42). Although they both possess a somewhat shorter scopula, both *Austropurcellia finniganensis* sp. n. and *Austropurcellia riedeli* sp. n. (blue and green points, respectively, on Fig. 2) have the same scopula orientation and scopula placement within the anal plate as other northernmost WT species (Fig. 42A, C). *Austropurcellia fragosa* sp. n. (white points on Fig. 2) has a unique scopula and anal plate shape that distinguishes it from all other *Austropurcellia* species found in the WT (Fig. 42B). Within species found further south in the rest of the WT, there is much greater variation in scopula morphology (Fig. 43, 44). However, the scopula emerges from the center or near the anterior margin of the anal plate in all of these species, distinguishing them from the northernmost WT species. One exception is a *Austropurcellia nuda* sp. n., (blue points on Fig. 3) which lacks a scopula on its anal plate (Fig. 32B), a trait that it shares only with *Austropurcellia absens*.

While this pattern of geographic distributions of shared characters states suggests closer relationships of geographically proximate species, an alternative interpretation is that unrelated species in certain regions of the WT have experienced morphological convergence. However, Boyer et al. (2015, 2016) found a distinct correlation between phylogenetic position within *Austropurcellia* and geographic proximity of species’ ranges, as inferred from a four-gene phylogeny of the genus; species that formed monophyletic or paraphyletic groups in the phylogeny were also recovered in close proximity to one another on distribution maps. Distinct geographic and phylogenetic groups emerged from the northernmost WT, the north-central WT, the central WT uplands, and the southern WT (Boyer et al. 2015) (Figs 1–4). Based upon these trends reported by Boyer et al. (2015), we postulate that geographic distance serves as a generally reliable proxy for phylogenetic affinity in *Austropurcellia*. Using distribution maps of the new species described here, we predict below the putative clades to which the new species described herein would belong, and used the distribution of shared morphological character states as a corroborative litmus test for inferred relationships.

Two of the new species described here have already been included in a recent molecular phylogenetic analysis (Boyer et al. 2016). One of them, *Austropurcellia megatanka* sp. n., is found at the top of Mt. Baldy as well as several localities within the Lamb Range including Mt. Tiptree, Mt. Haig, and a CSIRO trail in Danbulla State Forest (Fig. 4). These sites in the center of the Atherton Tablelands place *Austropurcellia megatanka* sp. n. in the northern end of the central WT uplands region (Fig. 4), suggesting that its closest relatives should include *Austropurcellia daviesae*, *Austropurcellia cadens*, and *Austropurcellia tholei*. Apropos, *Austropurcellia megatanka* sp. n. is similar to these species with regard to overall body shape and degree of tarsus IV bisegmentation, with its unusually long and wide scopula being its main distinguishing feature (Figs 19, 20, 44). Boyer et al. (2016) found that *Austropurcellia megatanka* (identified as “*Austropurcellia* sp. n. Baldy Mt.) is indeed a member of a clade that also includes *Austropurcellia daviesae*, *Austropurcellia cadens*, and *Austropurcellia tholei*.

The other species whose phylogenetic relationships have already been investigated is *Austropurcellia monteithi* sp. n., which is known from five localities throughout the Lamb Range (Davies Creek, Chujeba Peak summit, Mt. Edith summit, Mt. Williams summit, and the Kahlpahlim Rock trail), geographically placing this species in the north-central WT (Fig. 3). Boyer et al. (2016) found that this species (identified as “*Austropurcellia* sp. n. Lamb Range”) is related, as we would expect based on geography and morphology, as a member of a clade that also includes *Austropurcellia culminis*, *Austropurcellia scoparia*, and *Austropurcellia vicina. Austropurcellia nuda* sp. n. is found from only two localities (Black Mtn. and Black Mtn. summit), both located in the center of the distribution of this same north-central WT group (Fig. 3). We expect that it, too, is a close relative of *Austropurcellia culminis*, *Austropurcellia scoparia*, and *Austropurcellia vicina*. Both of these new species share morphological features such as body shape and adenostyle morphology with the north-central WT group, with the main exception again being differences in the scopula and anal plate. *Austropurcellia monteithi* sp. n. has a very long and wide scopula and *Austropurcellia nuda* sp. n. lacks a scopula entirely, both in contrast to the relatively narrow, short scopula of *Austropurcellia vicina* and *Austropurcellia culminis* and the very unusual scopula of *Austropurcellia scoparia*, which originates from the anterior margin of the anal plate (Figs 25, 26, 31, 32, 43).

*Austropurcellia finniganensis* sp. n., *Austropurcellia fragosa* sp. n., and *Austropurcellia riedeli* sp. n. all have distributions in the northernmost WT (Fig. 2). *Austropurcellia finniganensis* sp. n. is found in two localities on Mt. Finnigan, *Austropurcellia fragosa* sp. n. is found in three very proximate localities by Roaring Meg Creek as well as a locality in the McDowall Range, and *Austropurcellia riedeli* sp. n. was collected from a single locality along the Rossville-Bloomfield Road (Fig. 2). This suggests that these three species may be closely related to the other northernmost WT species such as *Austropurcellia articosa*, *Austropurcellia giribeti*, *Austropurcellia forsteri* Juberthie, 2000 and *Austropurcellia sharmai*, which have been found to form a paraphyletic grade at the base of the WT endemic clade within *Austropurcellia* (Boyer et al. 2015). This prediction is partially supported by morphology; *Austropurcellia finniganensis* sp. n., *Austropurcellia riedeli* sp. n. and *Austropurcellia fragosa* sp. n. all share morphological features such as body shape with *Austropurcellia sharmai* and *Austropurcellia forsteri*, but they lack the more pointed, elongated body shape shared by *Austropurcellia arcticosa* and *Austropurcellia giribeti*. *Austropurcellia finniganensis* sp. n., *Austropurcellia fragosa* sp. n., and *Austropurcellia riedeli* sp. n. also share another trait that central WT species lack—a very defined, ungranulated dorsal medial sulcus (Figs 7, 8, 13, 14, 37, 38, 42).

The WT is an important system for studying patterns and causes of rainforest endemism in both vertebrates and invertebrates. Yeates et al. (2002) investigated patterns and levels of endemism in 274 flightless insects at the scale of the 23 subregions within the WT (as defined by Winter 1984 and Williams and Pearson 1997). They found that 50% of species were endemic not only to the WT, but also to a single subregion within the WT, compared to only 15% subregional endemism within WT vertebrates (Yeates et al. 2002). Four subregions were found to contain the highest levels of subregional endemism in flightless insects: Finnigan Uplands, Carbine Uplands, Bellenden Ker/Bartle Frere Uplands, and Atherton Uplands (Yeates et al. 2002). When distributional data for the new species described here are combined with unpublished data collected in the lab of author SLB, we find that *Austropurcellia* conforms to the patterns found in other small flightless arthropods, with 50% of species endemic to single subregions. Only two of the 23 subregions contain more than one subregional endemic mite harvestman: Atherton Uplands and Finnigan Uplands. Of the six new species presented here: *Austropurcellia finniganensis* sp. n. and *Austropurcellia riedeli* sp. n. are both from the Finnigan Uplands, and indeed all of the species except for *Austropurcellia megatanka* are subregional endemics. Williams and Pearson (1997) articulated the hypothesis that the distribution of diversity and endemism across WT subregions could be explained by differential extinction during Pleistocene glacial cycling, when rainforest persisted in some subregions but was extirpated from others. Work in the lab of SLB modeling historical distribution of climatic conditions suitable for *Austropurcellia* from the Last Glacial Maximum to the present confirms that this pattern holds for our system (Boyer et al. 2016).

These predictions, and the postulated covariance of geographic distance, morphology, and phylogenetic relatedness in Cyphophthalmi more broadly, should be tested in the future using multilocus molecular phylogenies including the new species of *Austropurcellia* described herein. Such an approach would enable quantification of phylogenetic signal inherent to male morphological characters such as scopula and adenostyle shape, toward integrative taxonomy of mite harvestmen. Two putative new species from the WT remain undescribed due to incomplete collections and a lack of sufficient data, both in terms of morphology and DNA; however, we are confident that the majority of Queensland’s mite harvestmen diversity has now been documented. As we continue to approach complete species-level sampling of *Austropurcellia*’s extant diversity, we anticipate this genus will serve as robust model system to test hypotheses of how climatic and geologic changes in the WT have affected the distribution of genetic diversity, and how such processes leave their signature in the evolutionary history of Queensland’s paleoendemic fauna.

## Supplementary Material

XML Treatment for Austropurcellia

XML Treatment for Austropurcellia finniganensis

XML Treatment for Austropurcellia fragosa

XML Treatment for Austropurcellia megatanka

XML Treatment for Austropurcellia monteithi

XML Treatment for Austropurcellia nuda

XML Treatment for Austropurcellia riedeli
